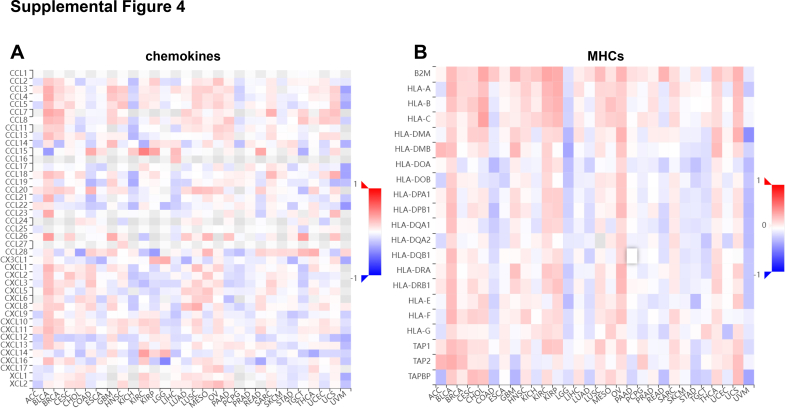# Exploring CISD1 as a multifaceted biomarker in cancer: Implications for diagnosis, prognosis, and immunotherapeutic response

**DOI:** 10.1016/j.gendis.2025.101677

**Published:** 2025-05-08

**Authors:** Caiyue Li, Zhipin Liang, Gabrielle Vontz, Connor Kent, Wenbo Ma, Lei Liu, Riya Dahal, Jovanny Zabaleta, Guoshuai Cai, Jia Zhou, Huangen Ding, Qiang Shen

**Affiliations:** a Department of Interdisciplinary Oncology, School of Medicine, LSU LCMC Cancer Center, Louisiana State University Health Sciences Center, New Orleans, LA 70112, USA; b Genetics Graduate Program, School of Medicine, LSU LCMC Cancer Center, Louisiana State University Health Sciences Center, New Orleans, LA 70112, USA; c Department of Pathology Laboratory Medicine, School of Medicine, Tulane University, New Orleans, LA 70112, USA; d IDP Graduate Program, School of Medicine, LSU LCMC Cancer Center, Louisiana State University Health Sciences Center, New Orleans, LA 70112, USA; e Department of Surgery, Colleges of Medicine and Public Health and Health Professions, University of Florida, Gainesville, FL 32611, USA; f Department of Biostatistics, Colleges of Medicine and Public Health and Health Professions, University of Florida, Gainesville, FL 32611, USA; g Department of Pharmacology and Toxicology, University of Texas Medical Branch, Galveston, TX 77555, USA; h Department of Biological Sciences, BMB Division, Louisiana State University, Baton Rouge, LA 70803, USA

**Keywords:** CISD1, Diagnostic biomarker, Immunotherapeutic biomarker, MitoNEET, Pan-cancer analysis, Prognostic biomarker

## Abstract

CISD1, an outer mitochondrial membrane iron-sulfur cluster protein, regulates intracellular iron levels, oxidative stress, and mitochondrial dynamics, playing critical roles in cellular bioenergetics and redox homeostasis. Although CISD1 has been identified as a prognostic biomarker in specific cancers, its broader implications in tumorigenesis, cancer progression, and immunotherapy remain unclear. Given the heterogeneity of cancer and the need for robust biomarkers across cancers, this study conducts the first comprehensive pan-cancer analysis of CISD1 by evaluating its roles in cancer and treatment. We obtained and analyzed data from databases including TCGA, GTEx, THPA, GEPIA2.0, SangerBox, cBioPortal, TIMER2.0, CAMOIP, DAVID, SRPLOT, and TISIDB. Our findings reveal significant alterations in CISD1 expression at both transcriptional and translational levels, as well as gene mutations across multiple cancers, indicating its potential as a diagnostic biomarker and its involvement in cancer development and progression. CISD1 dysregulation is linked to poor clinical outcomes, as shown through its impact on patient prognosis. GO and KEGG analyses show that CISD1 plays critical roles in cellular bioenergetics. Notably, CISD1 expression is significantly correlated with tumor stemness indices, tumor mutation burden, microsatellite instability, and immune checkpoint proteins in multiple cancers, and altered CISD1 levels are also observed in patients responding to immunotherapy, further supporting its role not only in prognosis but also as a key predictor in immunotherapy responses and outcomes. Our findings demonstrate CISD1 as a reliable and promising diagnostic, prognostic, and immunotherapeutic biomarker for multiple cancers, emphasizing its crucial role in cancer biology and potential to guide personalized cancer therapies.

## Introduction

Despite significant advances made in diagnosis and treatment in the past decades, cancer remains a major health challenge and the leading cause of mortality worldwide.^1^ The heterogeneous nature of cancer necessitates the identification of robust biomarkers for early detection, prognosis, and treatment response across multiple cancer types.^2^ Such biomarkers can be classified into diagnostic biomarkers, prognostic biomarkers, and/or predictive biomarkers.^2^ Diagnostic biomarkers help in the early detection of cancer, whereas prognostic biomarkers predict the likely course of diseases, and predictive biomarkers forecast the response to specific therapies.^2^ Current cancer biomarkers typically include genetic mutations such as breast cancer susceptibility gene 1/2 (BRCA1/2) in breast cancer,^3^ protein expression levels such as caveolin-1 in gastrointestinal cancers,^4^ and circulating tumor cells in hepatocellular carcinoma^5^ or DNA (ctDNA) in the bloodstream.^6^ However, there is still an urgent unmet need for novel and more effective biomarkers to enhance the accuracy and efficacy of cancer diagnosis and treatment.

The CDGSH iron sulfur domain (CISD) gene family encodes a group of NEET proteins (mitoNEET, NAF-1, and MiNT)^7^ that are a novel class of iron-sulfur ([2Fe–2S]) proteins characterized by a unique CDGSH domain, which binds to their respective [2Fe–2S] clusters.^8^ This domain gives NEET proteins distinctive properties, such as redox activity and lability of the [2Fe–2S] clusters, due to their unique coordination structure (3Cys:1His).^8^^,^^9^ This family includes three members: CISD1 (mitoNEET), CISD2 (NAF-1), and CISD3 (MiNT).^7^ MitoNEET (CISD1) is located on the outer mitochondrial membrane, and its unique [2Fe–2S] clusters endow mitoNEET with critical redox and pH-sensing properties, enabling it to maintain iron and redox homeostasis in the mitochondria.^8^^,^^10^ Nutrient-deprivation autophagy factor-1 (NAF-1/CISD2) is found in the outer mitochondrial membrane, endoplasmic reticulum, and mitochondria-associated membranes, and it links mitochondrial and endoplasmic reticulum functions, contributing to calcium signaling, autophagy, and apoptosis regulation; dysregulation of CISD2 is implicated in diseases like Wolfram syndrome and several cancers.^8^ Miner2 (CISD3) is localized in the mitochondrial matrix and primarily regulates mitochondrial iron-sulfur cluster biogenesis and respiratory chain function. Its role in cancer and other pathologies remains less defined compared with CISD1 and CISD2.^8^ Among the CISD family, CISD1 holds a distinct position due to its involvement in regulating mitochondrial dynamics, bioenergetics, ferroptosis, and apoptosis, playing a key role in cellular metabolism and oxidative stress response.^8^^,^^10^ The ability of the CISD1 protein to modulate mitochondrial iron metabolism and reactive oxygen species production suggests its significant influence on cellular function and survival. Intriguingly, our prior and current studies show that CISD1 functions as a novel redox enzyme to enhance oxidation of NADH and enlarge the NAD^+^ pool in the cytoplasm,^11^ possibly leading to aberrantly increased glycolysis and ATP/energy production. The role of CISD1 is even more important in cancers, as cancer cells often exhibit alterations in mitochondrial function to support rapid proliferation and resistance to apoptosis. Actually, studies have shown that CISD1 promotes breast cancer survival and proliferation by protecting the mitochondria of cancer cells from iron overaccumulation, enhancing the tolerance of cancer cells to oxidative stress, and suppressing autophagy and apoptosis.^8^ We speculated that by enhancing energy production and glycolysis in somatic cells by enhancing oxidation of NADH and the FMNH2/NADH energy production pathway/axis, CISD1 may act like an oncogene to promote malignant transformation and carcinogenesis.^12^ The role of CISD1 in cancer was first shown in breast cancer, where CISD1 is overexpressed and suppression of CISD1 significantly reduces cell proliferation and tumor growth,^13^^,^^14^ and it has been further identified as a novel target for breast cancer chemotherapy.^15^ In addition to breast cancer, CISD1 has been demonstrated as a potential novel antileukemic drug target for refractory or relapsed B-cell acute lymphocytic leukemia.^16^ These reports indicate CISD1's promising role in cancer treatment.

Although CISD1 has been identified as a prognostic biomarker in several cancers, such as breast cancer,^17^^,^^18^ hepatocellular carcinoma,^19^ gastric cancer,^20^ and bladder cancer,^21^ there is still a lack of systematic pan-cancer analysis of CISD1, which is vital to the understanding of its broad implications in cancer biology and clinical oncology. This manuscript aims to elucidate the expression patterns, genomic alterations, stemness profiles, and clinical significance of CISD1 across multiple cancer types. Using large-scale genomic datasets and advanced bioinformatics tools, we provide insights into the potential of CISD1 as a diagnostic, prognostic, and immunotherapeutic biomarker. Our results indicate that CISD1 expression correlates with patient prognosis and immune cell infiltration in various cancers. This pan-cancer approach highlights the universal and specific roles of CISD1, supporting its candidacy as a robust biomarker for predicting cancer outcomes and tailoring immunotherapy strategies. The comprehensive analyses presented in this study establish the significance of CISD1 in cancer and its potential value as a multi-biomarker for improving cancer prognosis and treatment.

## Materials and methods

### CISD1 gene and protein expression analysis

The mRNA expression data of CISD1 in 36 types of human normal tissues from the Genotype Tissue Expression (GTEx) cohort^22^ and 17 types of tumor tissues from The Cancer Genome Atlas (TCGA) cohort were downloaded from The Human Protein Atlas (THPA) online website.^23^ Specifically, the data was accessed by navigating to the “DATA” section of the website, where the relevant RNA expression dataset was identified and subsequently downloaded from the “DOWNLOADABLE DATA” section, and the data distribution was visualized using GraphPad Prism (version 10). The Gene Expression Profiling Interactive Analysis 2.0 (GEPIA2) is a web server for gene expression analysis based on tumor and normal samples from the TCGA and the GTEx databases; it accesses and processes data using R and Perl scripts,^24^ so we used GEPIA2 to visualize the different mRNA expression level of CISD1 across 33 TCGA tumor tissues versus their normal tissues (TCGA normal tissues combined with GTEx^22^ normal tissues). Briefly, by navigating to the “Expression Analysis” section and selecting “Expression Profile”, the gene CISD1 was searched in the “Gene” bar. Subsequently, data for all 33 cancer types were selected to obtain the results. The SangerBox portal (version 3.0) is a web-based tool platform that integrates GEO, TCGA, ICGC, and other databases and processes data in batches.^25^ We used this web tool to obtain and visualize the mRNA expression levels of CISD1 in different cancer stages, and in male/female patients of various cancers. The image staining results of protein expression of CISD1 in normal/cancer tissues were obtained from THPA in the “TISSUE"/"CANCER” section.

### Genetic alteration analysis

Characteristics of CISD1 genetic alterations were explored using the online cBio Cancer Genomics Portal (cBioPortal) database.^26^ Briefly, by selecting the “Quick Search” section, mutation type, alteration frequency, and copy number alteration data of CISD1 were obtained across the TCGA tumor datasets in the “Cancer Types Summary” module, and mutation types, sites, and numbers were obtained in the “Mutations” module. Genetic mutation ratios and mutation types of CISD1 in different tumors were obtained via the “Gene_Mutation” module on Tumor Immune Estimation Resource 2.0 (TIMER2)^27^ and via SangerBox, respectively. The mRNA expression values of CISD1 and its corresponding copy-number alteration values were analyzed using the SangerBox portal. Correlations of CISD1 expression and somatic mutations were obtained from the Comprehensive Analysis on Multi-Omics of Immunotherapy in Pan-cancer (CAMOIP)^28^ by selecting the “Mutational Landscape” section.

### Survival prognosis analysis

Overall survival, disease-specific survival, disease-free survival, and progression-free survival of CISD1 in various tumor types in the TCGA database were evaluated using forest plots by SangerBox online tools according to the website instructions. Overall survival map and the Kaplan–Meier survival plots were generated using GEPIA2 by selecting the “Survival Analysis” and “Survival Map” sections. High-expression and low-expression cohorts of CISD1 were obtained through the expression threshold of the cutoff-high (25%) and cutoff-low (75%) values.

### Stemness indices correlation analysis

Values of the correlation coefficient (Pearson's R) between CISD1 and stemness indices, including DNAss, EREG-METHss, DMPss, ENHss, RNAss, or EREG.EXPss were calculated and downloaded from the SangerBox portal, and the radar charts were generated using Microsoft Excel (Office 365). Correlations between CISD1 and RNA modifications were evaluated, and the chart was generated using Pearson's R by the SangerBox portal.

### CISD1 coexpression gene enrichment analysis

CISD1 coexpression genes and their corresponding Pearson coefficient were downloaded from cBioPortal by selecting the “Co-expression” section. CISD1 positively correlated genes with Pearson's *r* > 0.3 were selected to perform Gene Ontology (GO) term enrichment and Kyoto Encyclopedia of Genes and Genomes (KEGG) enrichment analysis using DAVID.^29^ Briefly, by navigating to the “Start Analysis” section, uploading and submitting the positive gene list, then selecting “Functional Annotation Tool”, the GO and KEGG gene cluster results were downloaded from the website. Venn diagram, GO Chord, and GO/KEGG pathway were visualized using Science and Research Online Plot (SRPLOT),^30^ which is an easy-to-use web server integrating more than a hundred commonly used data visualization and graphing functions together. GEPIA2 was used to visualize the heatmap of CISD1-positive coexpression genes by selecting the “Multiple Genes Comparison” section. Correlations between CISD1 and age were evaluated in the SangerBox portal according to the website instructions.

### Immune infiltration and immune-related genes analysis

Using Person's R correlations of CISD1 expression and StromalScore, ImmuneScore, ESTIMATEscore, or the proportion of tumor infiltration immune cells, including CD4^+^ T cells, CD8^+^ T cells, B cells, neutrophils, macrophages, and dendritic cells in tumors across cancer types, were assessed via the SangerBox portal. Correlation coefficient (Pearson's R) between CISD1 expression and tumor mutation burden (TMB), microsatellite instability (MSI), or neoantigens was downloaded from the SangerBox portal. Radar charts were generated using Microsoft Excel. Also, correlations between CISD1 expression and immune regulation genes, including immunoinhibitor and immunostimulator in different tumors from TCGA cohorts, were assessed via the SangerBox portal. In addition, correlations between CISD1 and chemokine or major histocompatibility complex (MHC) were assessed in TISIDB,^31^ which is a user-friendly web portal integrating multiple types of data resources in oncoimmunology by selecting the “Chemokine” and “Immunomodulator” sections. Expression levels of CISD1 in immunotherapy responders and non-responders were also obtained from TISIDB by selecting the “Immunotherapy” section.

### Statistical analysis

Hazard ratio (HR) and *p*-value were used to evaluate the significance of differences in survival analyses. Pearson's correlation coefficient and statistical significance were used to assess associations of gene expression, with the absolute value used to determine the strength of correlation. These results were considered statistically significant at ∗*p* < 0.05, ∗∗*p* < 0.01, ∗∗∗*p* < 0.001, and ∗∗∗∗*p* < 0.0001.

## Results

### Expression of CISD1 is up-regulated in multiple cancer types

Gene expression alterations often occur in the development and progression of different cancers. CISD1 was reported to be up-regulated in breast cancer and acute lymphoblastic leukemia.^13^^,^^15^^,^^16^ We therefore wondered if its expression was also altered across cancer types. We downloaded CISD1 mRNA expression data in different human normal tissues from GTEx datasets and in different tumor tissues from TCGA datasets via the THPA online website and analyzed the CISD1 mRNA expression level. In normal tissues, CISD1 had the highest mRNA expression level in skeletal muscle, followed by heart muscle, cerebral cortex, hypothalamus, *etc*. (Fig. 1A), suggesting that CISD1 may have a fundamental role in normal physiology in these tissues and organs. In tumor samples, it had the highest mRNA expression level in renal cancer, followed by colorectal cancer, cervical cancer, stomach cancer, *etc*. (Fig. 1B). Next, we wondered how many tumor tissues showed higher mRNA expression levels of CISD1 compared with corresponding normal tissues. Using GEPIA 2.0 online tools, we analyzed the mRNA expression levels of CISD1 in 33 tumor tissues compared with their corresponding normal tissues (TCGA normal tissue + GTEx normal tissues). We found that the mRNA expression level of CISD1 was prevalently up-regulated in 22 tumor tissues including breast invasive carcinoma (BRCA), cervical squamous cell carcinoma and endocervical adenocarcinoma (CESC), cholangiocarcinoma (CHOL), colon adenocarcinoma (COAD), lymphoid neoplasm diffuse large B-cell lymphoma (DLBC), esophageal carcinoma (ESCA), head and neck squamous cell carcinoma (HNSC), kidney chromophobe (KICH), kidney renal papillary cell carcinoma (KIRP), brain lower grade glioma (LGG), liver hepatocellular carcinoma (LIHC), lung adenocarcinoma (LUAD), lung squamous cell carcinoma (LUSC), ovarian serous cystadenocarcinoma (OV), pancreatic adenocarcinoma (PAAD), prostate adenocarcinoma (PRAD), rectum adenocarcinoma (READ), skin cutaneous melanoma (SKCM), stomach adenocarcinoma (STAD), thymoma (THYM), uterine corpus endometrial carcinoma (UCEC), and uterine carcinosarcoma (UCS) (Fig. 1C), suggesting an oncogenic role for CISD1 in these cancers. Conversely, CISD1 expression was down-regulated in 6 tumor tissues: bladder urothelial carcinoma (BLCA), glioblastoma multiforme (GBM), acute myeloid leukemia (LAML), sarcoma (SARC), testicular germ cell tumors (TGCT), thyroid carcinoma (THCA) (Fig. 1C), suggesting that CISD1 may play tumor suppressive functions in these cancers. CISD1 showed no alteration of mRNA expression level in 5 tumor tissues: adrenocortical carcinoma (ACC), kidney renal clear cell carcinoma (KIRC), mesothelioma (MESO), pheochromocytoma and paraganglioma (PCPG), uveal melanoma (UVM) (all abbreviations are showed in Table S1) (Fig. 1C). Furthermore, from SangerBox online tools analysis, we found a gradual increase in CISD1 mRNA levels across different cancer stages, from stage I to stage IV, in LUAD, LUSC, THYM, and LIHC (Fig. S1A), further confirming the possibility that CISD1 plays an oncogenic role and is associated with cancer progression in these cancers. In addition, CISD1 may be important for metastasis in certain cancer types, as we found that CISD1 mRNA levels were increased in the M1 stage when compared with the M0 stage in BRCA and KIRC (Fig. S1B), and increased across different lymph node stages, from stage N0 to stage N3, in LUAD, BRCA, HNSC, LUSC, and READ (Fig. S1C); while decreased in COAD and THCA (Fig. S1C).

**Figure 1:**
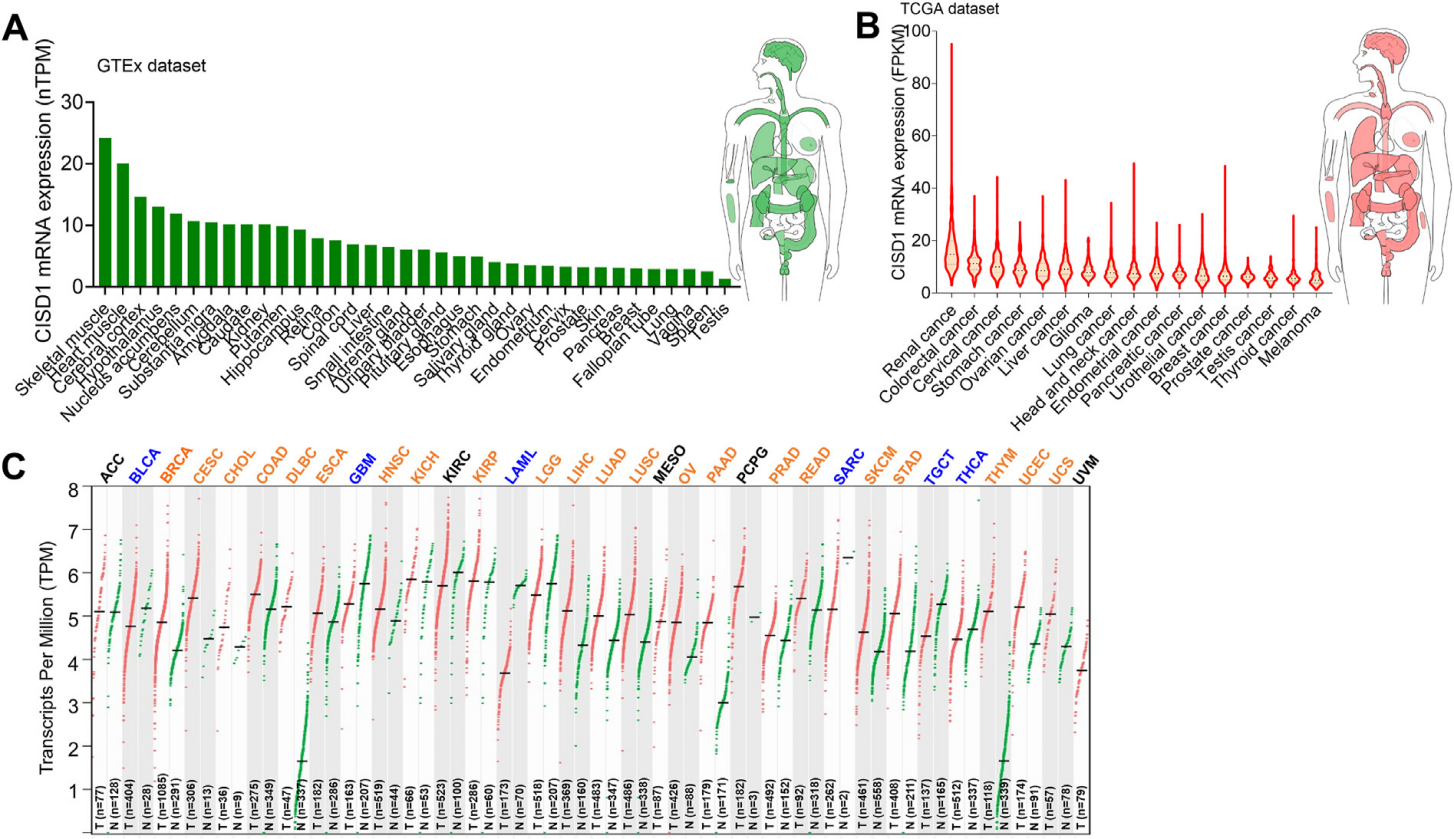
The mRNA expression levels of CISD1 in human pan-cancer. **(A)** CISD1 mRNA expression in various human normal tissues. Genotype Tissue Expression (GTEx) data for CISD1 mRNA in human normal tissues were downloaded from The Human Protein Atlas (THPA) and analyzed using GraphPad Prism. **(B)** CISD1 mRNA expression in various human cancer tissues. The Cancer Genome Atlas (TCGA) data for CISD1 mRNA expression across different cancer types were retrieved from THPA and analyzed using GraphPad Prism. **(C)** CISD1 mRNA expression levels in tumor tissues were compared with normal tissues across 33 cancer types. Differential expression analysis was conducted using GEPIA2, with a significance threshold of *Q* < 0.05 (Benjamini-Hochberg correction for multiple testing). Cancer types with significantly increased CISD1 expression are indicated in brown font, while those with significantly decreased expression are in blue font. N = normal tissue, and T = tumor tissue.

Next, we wondered if the protein expression level of CISD1 was also increased in various cancers. We downloaded the protein staining images from THPA, and found that the protein level of CISD1 was significantly higher in 14 cancers compared with their corresponding normal tissues including breast cancer, cervix cancer, colorectal cancer, lymphoma, renal cancer, liver cancer, lung cancer, pancreatic cancer, prostate cancer, skin cancer, stomach cancer, testis cancer, endometrium cancer, and thyroid cancer (Fig. 2), which is consistent with the mRNA analysis in Figure 1. Taken together, these results reveal significant variations in CISD1 expression, with most cancers showing elevated levels. This suggests CISD1 may be involved in tumorigenesis and is a potential diagnostic biomarker for these cancer types. These findings further highlight the importance of conducting comprehensive pan-cancer analyses to uncover the broader implications of CISD1 in oncology.

**Figure 2:**
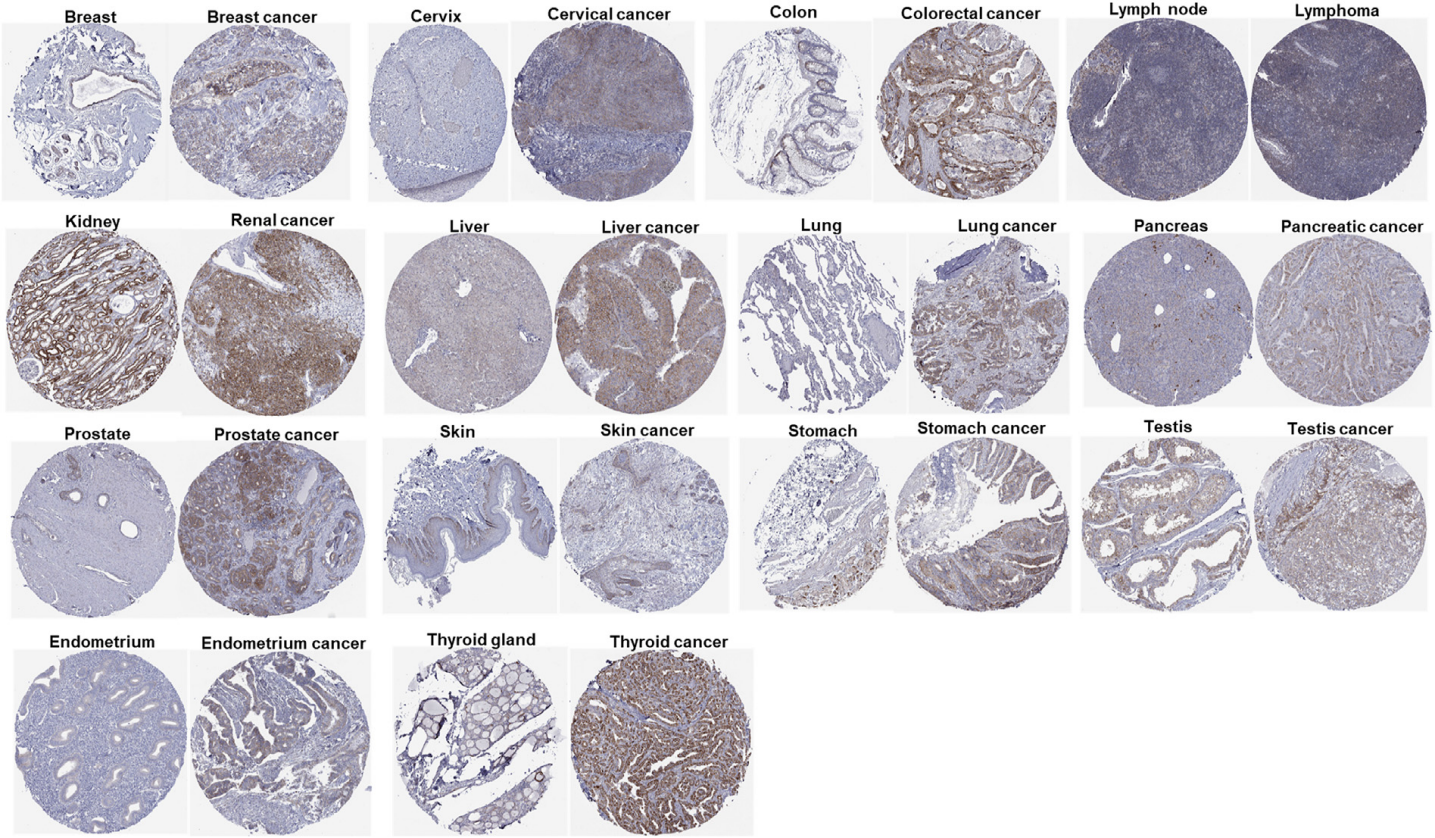
Protein expression levels of CISD1 in human pan-cancer. Immunohistochemical staining images show CISD1 protein expression in various normal human tissues and corresponding cancer tissues. The data for these images were downloaded from The Human Protein Atlas (THPA). For each type of tissue, the normal tissue is displayed on the left, and the corresponding cancer tissue is on the right. CISD1 protein was stained brown.

### Alterations in the CISD1 gene are associated with the development and progression of multiple cancer types

In addition to gene expression, cancer patient outcomes such as survival and disease recurrence are affected by gene alterations and mutations. Activation of oncogenes or inactivation of tumor suppressor genes drives the initiation and progression of cancer.^32^^,^^33^ Understanding such alterations helps in elucidating the mechanisms of cancer development and disease progression. We next analyzed the genetic profile of CISD1 in cancer tissues. Using cBioPortal, CISD1 showed mutation frequency in several cancers, in which UCEC (>1%) had the highest rate of CISD1 gene mutations frequency, followed by STAD, SARC, LIHC, SKCM, OV, and LUAD (Fig. 3A). Consistent with these results, we found that UCEC showed the highest percentage of patient samples with CISD1 mutations (0.0094%), followed by STAD (0.0046%), SARC (0.0042%), LIHC (0.0027%), OV (0.0024%), SKCM (0.0021%), and LUAD (0.0019%) from exploring CISD1 gene mutation rates on TIMER 2.0 website (Fig. 3B). Using SangerBox online analysis, we further explored the mutation types and mutation sites of CISD1 and found that missense mutations were the main type of CISD1 mutation in cancer (Fig. 3C). According to cBioPortal online analysis (Fig. 3D), the mutation sites frequently occur within the zf-CDGSH domain, which binds to iron to form a redox-active pH-labile 2Fe–2S cluster, and A69 S/V is a hot spot mutation site, suggesting that the mutations of CISD1, especially the hot spot A69 S/V, could be a diagnostic biomarker.

**Figure 3:**
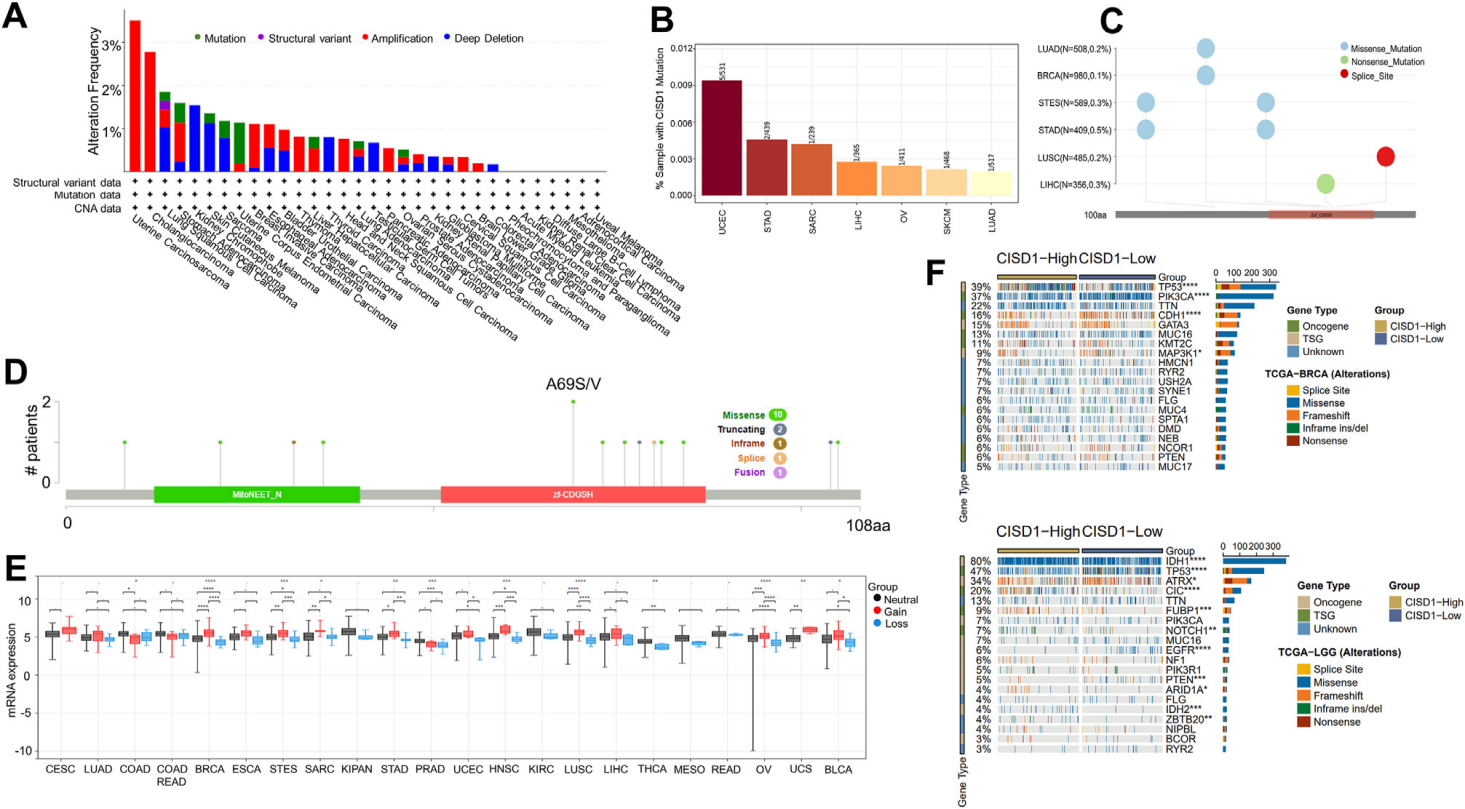
Distinct genomic profile analysis of CISD1 in various cancers. **(A)** Alteration frequency of CISD1 across various cancer types. The data were obtained from the cBio Cancer Genomics Portal (cBioPortal) database, showing the percentage of samples with mutations, structural variants, amplifications, and deep deletions in CISD1 across different cancer types. **(B)** Mutation frequency of CISD1 in different cancer types. The data from Tumor Immune Estimation Resource 2.0 (TIMER2.0) reveals the percentages of mutations of CISD1 in various cancers. **(C)** Mutation types and distribution in CISD1 across cancers. The data was analyzed using the SangerBox portal, showing the percentages of missense mutations, nonsense mutations, and splice sites across cancer types. **(D)** Protein domain analysis of CISD1 mutations. The mutation map generated from cBioPortal shows mutation types in the MitoNEET_N and zf-CDGSH domains. **(E)** mRNA expression analysis of CISD1 alterations in different cancer types. The results were obtained from SangerBox comparing neutral, gain, and loss alterations in various cancers. **(F)** The relationship between CISD1 expression and somatic mutations in BRCA (top) and LGG (bottom) cancers. The data from Comprehensive Analysis on Multi-Omics of Immunotherapy in Pan-cancer (CAMOIP) show the percentages of oncogene, tumor suppressor gene, and unknown gene types, and show the numbers of different mutation types such as splice site, missense, frameshift, inframe insertion/deletion, and nonsense mutations in the CISD1-high and CISD1-low groups.

In addition, cBioPortal analysis showed that UCS (>3%), CHOL (>2%), STAD (>1%), and BRCA (>1%) had a higher rate of CISD1 gene amplification frequency (Fig. 3A); while kidney chromophobe (>1%), SKCM (>1%), and lung squamous (>1%) had a higher rate of CISD1 gene deep deletion frequency (Fig. 3A). Further analysis utilizing SangerBox showed that gain copy number of CISD1 is associated with higher mRNA expression levels of CISD1 in 19 cancer types including CESC, LUAD, BRCA, ESCA, STES (Stomach and Esophageal carcinoma), SARC, KIPAN (Pan-kidney cohort (KICH + KIRC + KIRP), STAD, UCEC, HNSC, KIRC, LUSC, LIHC, THCA, MESO, READ, OV, UCS, BLCA (Fig. 3E), suggesting that CISD1 mRNA expression level is mainly due to the copy-number alterations.

Next, the relationship between CISD1 expression and specific genomic characteristics, such as somatic mutations, was analyzed using CAMOIP online analysis. We found that CISD1 was strongly associated with somatic mutations in multiple cancers. The CISD1-high group showed high frequency of somatic mutations in TP53 (39%), PIK3CA (37%), CDH1 (16%), MAP3K1 (9%) in BRCA (Fig. 3F, top), and IDH1 (80%), TP53 (47%), ATRX (34%), CIC (20%), FUBP1 (9%), NOTCH1 (7%), EGFR (6%), PTEN (5%) in LGG (Fig. 3F, bottom). Overall, these results show CISD1 gene mutations, amplifications, and deletions are present in multiple cancers, with missense mutations as the most frequent type of CISD1 gene mutations in various cancers, and copy number alterations as the most frequent genomic change in most of the cancers. Moreover, expression of CISD1 was strongly associated with somatic mutations. Taken together, these results suggest that CISD1 gene alterations may regulate the initiation, growth, and progression of various cancers and can serve as a potential diagnostic biomarker.

### CISD1 expression is associated with the prognosis of various tumors

**Figure 4:**
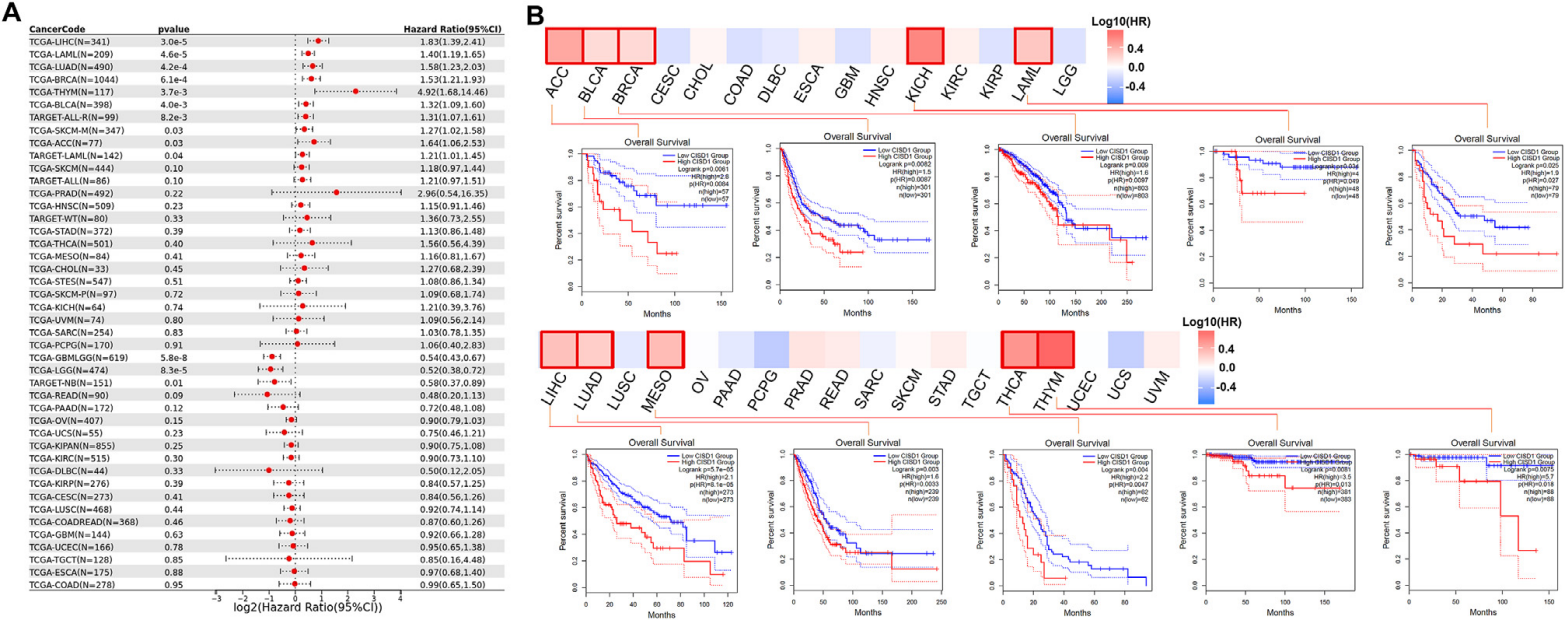
Correlation analysis between CISD1 expression and overall survival in different cancers. **(A)** The forest plot illustrates the hazard ratios (HR) of CISD1 expression in various cancers. The data were obtained from SangerBox and showed the correlation between CISD1 expression and overall survival across multiple cancer types. A *p*-value less than 0.05 is considered significant. **(B)** Survival map and Kaplan–Meier survival curves for overall survival in various cancer types. The results were generated using GEPIA2.0 online tools. The survival heatmap above represents log10(HR) values, with red indicating higher HR and blue indicating lower HR; cancer types highlighted with a red box on the heatmap indicate statistical significance, with a *p*-value less than 0.05. Blue and red lines in the Kaplan–Meier plots represent patients with low and high CISD1 expression, respectively. Log-rank *p*-values and hazard ratios (HR) are displayed for each plot.

Gene expression profiles can help identify biomarkers that predict patient outcomes, such as overall survival, disease-specific survival, disease-free survival, and progression-free survival.^34^ Our findings above render CISD1 a candidate for further investigation as a prognostic biomarker. Thus, we compared survival in cancer patients with high or low CISD1 expression levels. Using SangerBox online tools, we generated survival forest plots and found that higher CISD1 expression was significantly associated with worse patient overall survival in LIHC (HR = 1.83, *p* = 3.0e-5), LAML (HR = 1.40, *p* = 4.6e-5), LUAD (HR = 1.58, *p* = 4.2e-4), BRCA (HR = 1.53, *p* = 6.1e-4), THYM (HR = 4.92, *p* = 3.7e-3), BLCA (HR = 1.32, *p* = 4.0e-3), SCKM (HR = 1.27, *p* = 0.03), and ACC (HR = 1.64, *p* = 0.03) (Fig. 4A). In contrast, higher CISD1 expression was significantly associated with lower risk of death in GBMLGG (Glioma) (HR = 0.54, *p* = 5.8e-8), LGG (HR = 0.52, *p* = 8.3e-5), and NB (Neuroblastoma) (HR = 0.58, *p* = 0.01) (Fig. 4A). We also used GEPIA 2.0 online tools to generate survival heatmaps and Kaplan–Meier survival curves to reveal the relationship between CISD1 expression and overall survival across various cancer types. From the survival heatmaps and the corresponding Kaplan–Meier survival curves, high CISD1 expression is associated with worse patient survival (Fig. 4B) for ACC, HR = 2.8, *p* = 0.0061; BLCA, HR = 1.5, *p* = 0.0082; BRCA, HR = 1.6, *p* = 0.009; KICH, HR = 4, *p* = 0.02; LAML, HR = 1.9, *p* = 0.025; LIHC, HR = 2.1, *p* = 5.7e-05; LUAD, HR = 1.6, *p* = 0.003); MESO, HR = 2.2, *p* = 0.004; THCA, HR = 3.5, *p* = 0.0081; and THYM, HR = 5.7, *p* = 0.0075. Furthermore, disease-specific survival analysis showed that BLCA (HR = 1.45, *p* = 1.9e-3), LIHC (HR = 1.74, *p* = 2.5e-3), LUAD (HR = 1.59, *p* = 3.9e-3), BRCA (HR = 1.54, *p* = 0.01), ACC (HR = 1.65, *p* = 0.03) had significantly stronger association between CISD1 and patient survival (Fig. S2A) where patients with higher expression levels of CISD1 had a higher risk of death. Disease-free survival and progression-free survival analyses are essential tools for assessing treatment efficacy.^35^ Disease-free survival analysis revealed that LIHC patients had a significantly higher risk of death (HR = 1.40, *p* = 0.01) (Fig. S2B). Similarly, progression-free survival analysis indicated that patients with elevated CISD1 expression faced a heightened risk of death across multiple cancers: ACC (HR = 1.67, *p* = 5.1e-3), LIHC (HR = 1.35, *p* = 0.01), BLCA (HR = 1.28, *p* = 0.01), LUAD (HR = 1.33, *p* = 0.02), and SKCM (HR = 1.18, *p* = 0.05) (Fig. S2C), suggesting that the progression of the disease cannot be effectively controlled by the treatment of these cancers. In contrast, patients with higher CISD1 expression showed significantly lower risk of death in GBMLGG (Fig. S2A–C) and LGG (Fig. S2A–C) from disease-specific survival analysis, disease-free survival analysis, and progression-free survival analysis.

Although the results highlighted that CISD1 expression levels have varying impacts on overall survival across different cancer types, high CISD1 expression is generally associated with worse survival. Taken together, the significant associations between CISD1 expression and patient outcomes strongly suggest that CISD1 can be a potential prognostic biomarker for multiple cancers.

### CISD1 is positively correlated with cancer stemness and RNA modifications in multiple cancers

**Figure 5:**
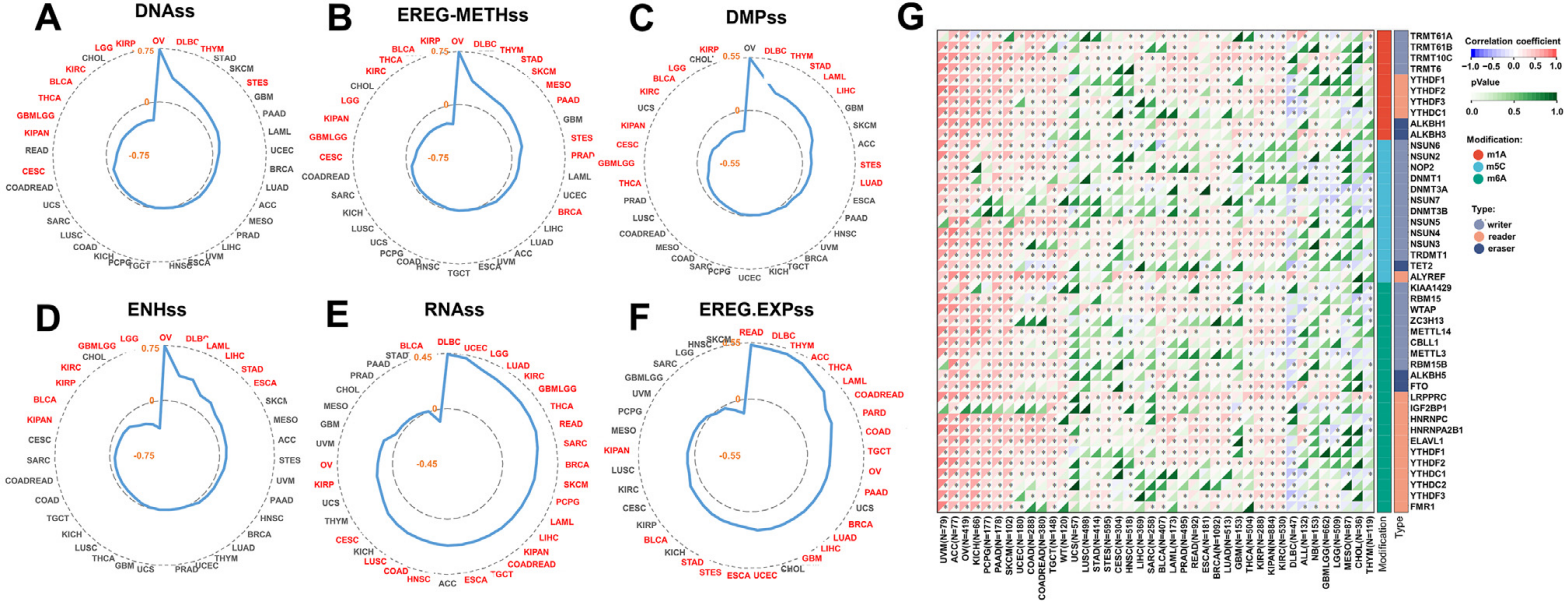
The correlation analysis between CISD1 and cancer stemness or RNA modifications. **(A**–**F)** The radar plots display the correlation between CISD1 expression and various cancer stemness scores in different cancers. The data were downloaded from SangerBox. The following stemness indices are presented: DNAss (A), EREG-METHss (B), DMPss (C), ENHss (D), RNAss (E), and EREG.EXPss (F). Each plot shows the correlation coefficient, with cancer types labeled around the plot perimeter. Cancer types with significant correlations (*p* < 0.05) are highlighted in red. The blue line represents the correlation values, with a negative correlation coefficient shown inside the plot. **(G)** The heatmap illustrates the correlation between CISD1 expression and various RNA modification-related genes (writers, readers, and erasers) across different cancer types. The data were presented as correlation coefficients, with colors ranging from red (positive correlation) to blue (negative correlation). The types of RNA modifications (m1A, m5C, and m6A) are indicated by the colored bars on the right. The *p*-values for each correlation are shown on the heatmap, with significant correlations highlighted by asterisks, and dark green means no significant correlation.

Cancer stem cells have the unique ability to self-renew and differentiate into various cell types that make up the tumor; these cells are thought to be responsible for tumor initiation, progression, therapeutic resistance, and relapse after treatment.^36^ Stemness indices, such as DNA methylation-based stemness score (DNAss), Differentially Methylated Position Stemness Score (DMPss), Enhancer Stemness Score (ENHss), RNA Stemness Score (RNAss), Epigenetically Regulated Gene Methylation Stemness Score (EREG-METHss), and Epigenetically Regulated Gene Expression Stemness Score (EREG.EXPss), quantify the stem-like characteristics of cancer cells, making them valuable tools for studying tumor biology.^37^^,^^38^ We assessed whether CISD1 is associated with these stemness indices. Using SangerBox online analysis tools, surprisingly, we found that CISD1 was significantly and positively correlated with cancer stemness indices: DNAss in OV, DLBC, THYM, STAD, and STES (Fig. 5A); EREG-METHss in OV, DLBC, THYM, STAD, SKCM, MESO, PAAD, STES, PRAD, and BRCA (Fig. 5B); DMPss in OV, DLBC, THYM, STAD, LAML, LIHC, STES, and LUAD (Fig. 5C); ENHss in OV, DLBC, LAML, LIHC, STAD, and ESCA (Fig. 5D). Intriguingly, CISD1 was significantly and positively correlated with RNAss in multiple cancer types including DLBC, UCEC, LGG, LUAD, KIRC, GBMLGG, THCA, READ, SARC, BRCA, SKCM, PCPG, LAML, LIHC, KIPAN, COADREAD (colon adenocarcinoma/rectum adenocarcinoma), TGCT, ESCA, HNSC, COAD, LUSC, CESC, KIRP, and OV (Fig. 5E); and EREG.EXPss in READ, DLBC, THYM, ACC, THCA, LAML, COADREAD, PRAD, COAD, TGCT, OV, PAAD, BRCA, LUAD, LIHC, GBM, UCEC, ESCA, STEX, STAD, BLCA, and KIPAN (Fig. 5F). These results suggest that higher CISD1 expression is associated with enhanced expression of stemness-related genes, likely contributing to maintaining or enhancing these stem-like properties. This association could explain its potential role in promoting tumor aggressiveness and therapeutic resistance in these cancers. Whereas, CISD1 showed a significant negative correlation with cancer stemness indices: DNAss in KIRP, LGG, KIRC, BLCA, THCA, GBMLGG, KIPAN, and CESC (Fig. 5A); EREG-METHss in KIRP, BLCA, THCA, KIRC, LGG, KIPAN, GBMLGG, and CESC (Fig. 5B); DMPss in KIRP, LGG, BLCA, KIRC, KIPAN, CESE, GBMLGG, and THCA (Fig. 5C); ENHss in LGG, GBMLGG, KIRC, KIRP, BLCA, and KIPAN (Fig. 5D); RNAss in BLCA (Fig. 5E). Overall, the association between CISD1 and cancer cell stemness varies by cancer type.

The results above show that CISD1 expression is linked to the methylation status of genes regulated by epigenetic mechanisms that contribute to cancer stemness, especially, higher CISD1 expression is associated with an RNA expression profile that resembles that of cancer stem cells, implying that CISD1 is associated with epigenetic modifications. Epigenetic modifications, such as RNA modifications, the new frontier of this arena, have significant implications for cancer progression and treatment.^39^ We next used SangerBox online tools to analyze the correlation between CISD1 mRNA expression and RNA modification enzymes that catalyze m1A, m5C, and m6A, as these RNA modifications play critical roles in cancer by regulating gene expression, mRNA stability, translation, and tumor progression.^39^ As shown in Figure 5G, CISD1 mRNA expression was significantly and positively correlated with m1A, m5C, and m6A related RNA modification enzymes in UVM, ACC, OV, KICH, PCPG, PAAD, SKCM, UCEC, COAD, COADREAD, TGCT, WT, STAD, STES, HNSC, LIHC, BLCA, LAML, PRAD, READ, ESCA, BRCA, LUAD, THCA, KIRP, KIRC, KIPAN, and KIRC, suggesting that m1A, m5C, and m6A RNA modifications may contribute to gene expression related to tumor progression in these cancers in which when CISD1 expression increases. In contrast, CISD1 mRNA expression was significantly and negatively correlated with m1A, m5C, and m6A related RNA modification enzymes in DLBC (Fig. 5G). Taken together, these results suggest that CISD1 could be a potential biomarker for identifying tumors with high stemness and may serve as a therapeutic target to inhibit these aggressive tumor characteristics.

### CISD1 coexpression gene enrichment analysis in various tumors

**Figure 6:**
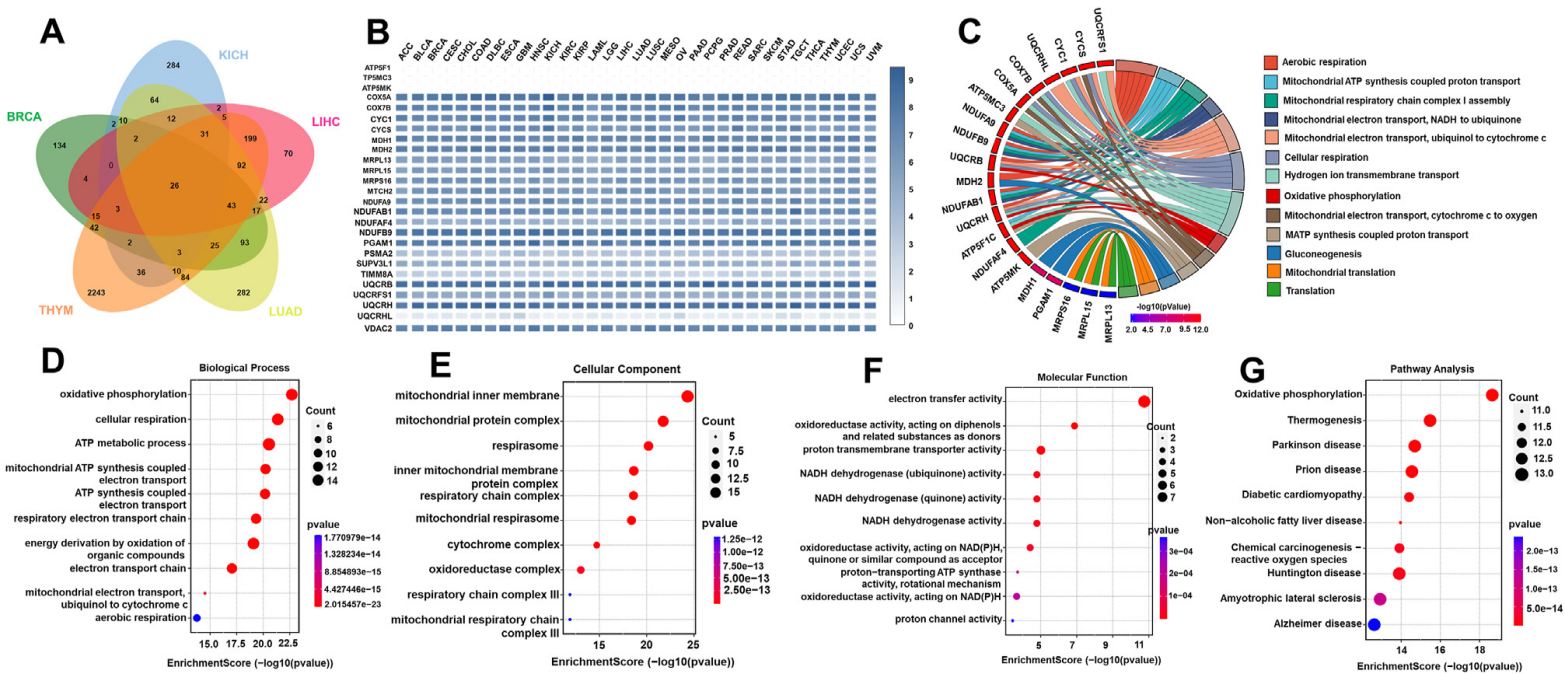
CISD1 coexpression gene enrichment analysis in various tumors. **(A)** A Venn diagram was generated from SangerBox showing the overlap of CISD1 coexpression genes in five different tumor types: BRCA (green), THYM (orange), LUAD (yellow), LIHC (red), and KICH (blue). The numbers inside the diagram indicate the count of common and unique genes coexpressed with CISD1 across these tumors. **(B)** A heatmap was generated from Gene Expression Profiling Interactive Analysis 2.0 (GEPIA2.0), representing the expression levels of the 26 CISD1 coexpressed genes across various cancers. The color gradient from light to dark blue indicates increasing levels of gene expression. **(C)** A Gene Ontology (GO) Chord plot was generated from Science and Research Online Plot (SRPLOT), displaying the enrichment of the 26 CISD1 coexpressed genes. Biological process pathways are highlighted with colored bands, with the width corresponding to the number of enriched genes. **(D**–**G)** The bubble plots were generated from SRPLOT, representing the gene ontology (GO) and Kyoto Encyclopedia of Genes and Genomes (KEGG) pathway enrichment analysis of the 26 CISD1 coexpressed genes in tumors. (D) Biological processes related to CISD1 coexpressed genes. (E) Cellular components associated with these genes. (F) Molecular functions of CISD1 coexpressed genes. (G) KEGG pathway analysis shows significant enrichment. Each bubble's size corresponds to the number of genes involved, while color intensity indicates the significance level (*P*).

To understand the biological significance of CISD1 and its coexpression genes and their roles within cellular processes, and to elucidate how CISD1 influences cancer progression and patient outcomes, we performed GO and KEGG enrichment analyses for CISD1 and its coexpression genes, as GO and KEGG analyses are crucial in understanding the biological significance of genes and their roles within cellular processes.^40^^,^^41^ Since BRCA, KICH, LIHC, LUAD, and THYM all showed higher mRNA expression levels of CISD1, high stemness signatures, and high levels of RNA modifications with worse patient survival from the results above, we wondered if there are overlapping CISD1 coexpression genes in these cancers, and if these genes are potentially involved in fundamental processes related to CISD1 in cancers. We first downloaded the CISD1-positive coexpression genes in these five cancers from cBioPortal, and performed Venn analysis using SangerBox online tools, and found that there were 26 overlapped genes in the five cancers analyzed (Fig. 6A). Next, we analyzed the mRNA expression levels of these 26 overlapped genes in different cancers, and the heatmap showed that 20 of these overlapped genes were highly expressed in different cancers (Fig. 6B), suggesting that these genes may play crucial roles in ontogenesis and cancer progression. From GO enrichment analysis, we found that these genes are involved in oxidative phosphorylation, cellular respiration, ATP metabolic process, mitochondrial ATP synthesis coupled electron transport, ATP synthesis coupled electron transport, respiratory electron transport chain, energy derivation by oxidation of organic compounds, electron transport chain, mitochondrial electron transport ubiquinol to cytochrome c, and aerobic respiration (Fig. 6C, D). They were mainly localized to mitochondria, respirasome, respiratory chain complex, cytochrome, and oxidoreductase complex (Fig. 6E), with molecular functions of electron transfer activity, oxidoreductase activity, proton transmembrane transporter activity, NADH dehydrogenase activity, proton-transporting ATP synthase activity, and proton channel activity (Fig. 6F). To further understand how these genes interact with networks and pathways, we also performed KEGG enrichment analysis. These CISD1 coexpressed genes were involved in thermogenesis, Parkinson's disease, prion disease, diabetic cardiomyopathy, non-alcoholic fatty liver disease, chemical carcinogenesis, reactive oxygen species, Huntington disease, amyotrophic lateral sclerosis, and Alzheimer's disease (Fig. 6G). Taken together, these results suggest that CISD1 coexpression genes are involved in vital biological processes, particularly related to cellular energy production and metabolic processes, and likely play significant roles in cancer cell survival and proliferation.

Interestingly, CISD1 mRNA expression levels were negatively correlated with age in CHOL, GBM, READ, CESC, PAAD, GBMLGG, TGCT, COADREAD, COAD, OV, BRCA, LGG, and LUSC (Fig. S3A), suggesting that younger patients have higher mRNA levels of CISD1 in these cancers, further supporting the above results that CISD1 is associated with bioenergetics. Male patients showed higher mRNA expression levels of CISD1 in LUAD, HNSC (Fig. S3B), while female patients showed higher mRNA expression levels of CISD1 in KIPAN and KIRC (Fig. S3B), suggesting that its expression pattern in cancer has limited gender preference.

### CISD1 regulates tumor infiltration of immune cells in multiple cancers

**Figure 7:**
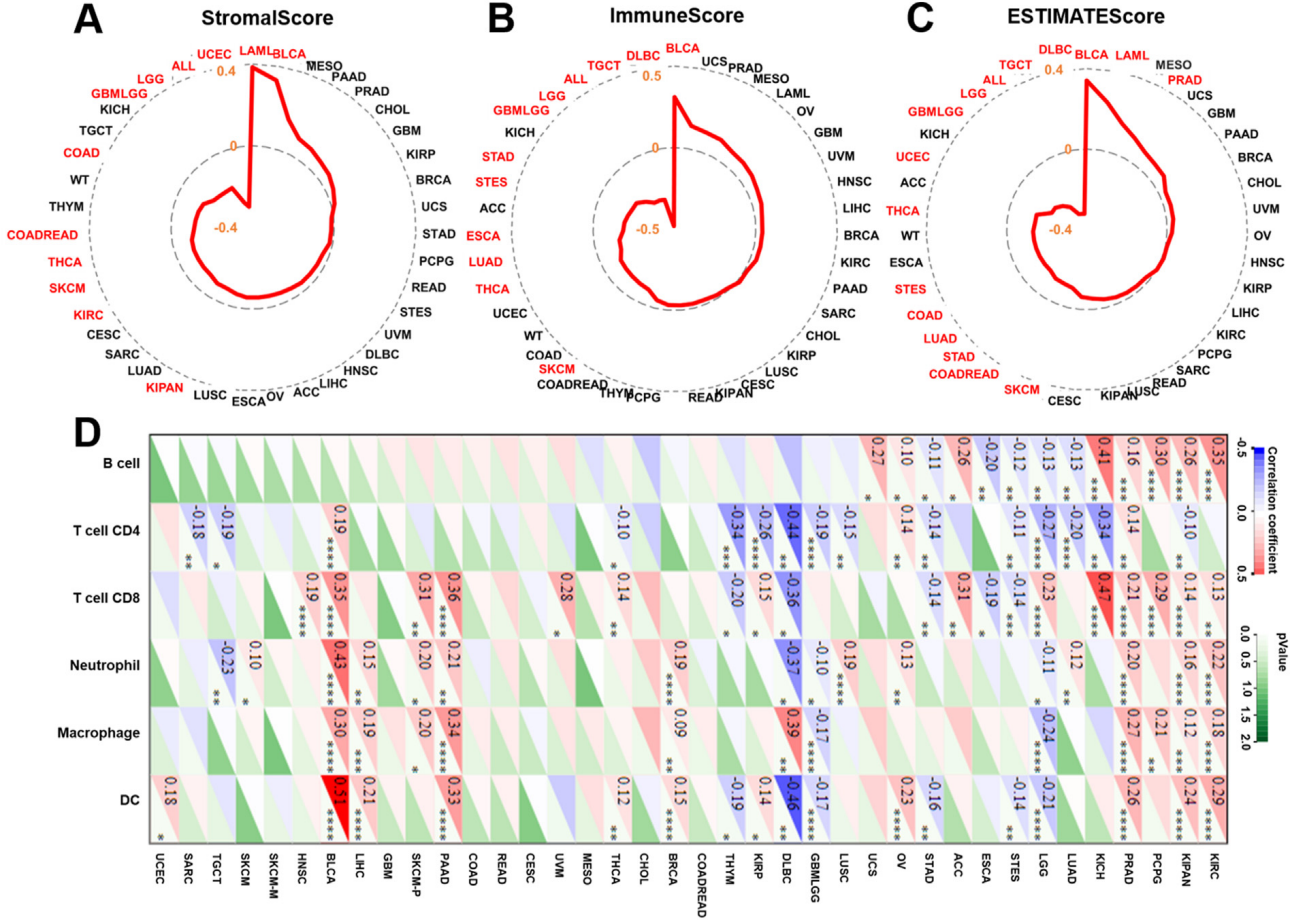
Correlation analysis between CISD1 and immune infiltration in multiple cancers. **(A**–**C)** The radar plots depict the correlation between CISD1 expression and various immune-related scores, including the StromalScore (A), ImmuneScore (B), and ESTIMATEScore (C) across different cancers. These analyses were performed using SangerBox. Each plot shows the correlation coefficients, with cancer types labeled around the perimeter. Significant correlations (*p* < 0.05) are marked in red. The red lines indicate the correlation levels, and the values of negative correlation coefficients are shown inside the plot. **(D)** The heatmap generated from SangerBox displays the correlation coefficients between CISD1 expression and the infiltration levels of various immune cell types, including B cells, T cells (CD4 and CD8), neutrophils, macrophages, and dendritic cells (DC), across multiple cancer types. Positive correlations are indicated in red, and negative correlations are in blue. The color intensity corresponds to the strength of the correlation, and significant correlations (*p* < 0.05) are indicated by asterisks, and dark green means no significant correlation.

The tumor microenvironment (TME) is composed of various immune cells, stromal cells, and extracellular components.^42^ Immune infiltration analysis helps in understanding the composition and functional state of these immune cells within the TME, and whether the TME of a tumor can promote or inhibit tumor growth and metastasis.^43^^,^^44^ StromalScore quantifies the presence of stromal cells, including fibroblasts, mesenchymal cells, and other connective tissue cells that support the structure of tissues within the tumor microenvironment. A high stromal content is often associated with more aggressive tumor behavior, resistance to therapy, and poorer prognosis^45^; ImmuneScore assesses the infiltration of immune cells, particularly lymphocytes like T cells, B cells, and natural killer (NK) cells et al in the tumor microenvironment, and a higher ImmuneScore is often associated with better responses to immunotherapy and better patient outcomes.^45^ An ESTIMATEscore is a composite score derived from the StromalScore and ImmuneScore, where a higher ESTIMATEscore indicates an immunotherapy could be less effective.^45^ We wondered if CISD1 is associated with immune infiltration and immunotherapy outcomes. Raw data were downloaded from SangerBox, and immune scores were analyzed. The results showed that CISD1 was significantly and positively correlated with StromalScore in LAML and BLCA, suggesting that patients with higher expression of CISD1 may be more resistant to therapies in these cancers; while CISD1 showed significantly negative correlation with StromalScore in UCEC, acute lymphocytic leukemia, LGG, GBMLGG, COAD, THCA, SKCM, KIRC, and KIPAN (Fig. 7A), suggesting that patients with higher CISD1 expression might respond better to certain therapies and have better prognoses in these cancers. CISD1 was also significantly and positively correlated with ImmuneScore in BLCA (Fig. 7B), suggesting that higher CISD1 expression is associated with greater immune cell infiltration in the TME; while CISD1 was significantly negatively correlated with ImmuneScore in DLBC, TGCT, acute lymphocytic leukemia, LGG, GBMLGG, STAD, STES, ESCA, LUAD, THCA, and SKCM (Fig. 7B), suggesting that higher CISD1 expression is associated with reduced immune cell infiltration in the TME. CISD1 showed a significant positive correlation with ESTIMATEscore in BLCA, LAML, and PRAD (Fig. 7C), suggesting that CISD1 may contribute to a dense and complex TME. In contrast, CISD1 showed significant negative correlation with ESTIMATEscore in DLBC, TGCT, acute lymphocytic leukemia, LGG, GBMLGG, UCES, THCA, STES, COAD, LUAD, STAD, COADREAD, and SKCM (Fig. 7C), suggesting that CISD1 may be involved in creating a less dense TME. Taken together, these results suggest that CISD1 may serve as a predictive factor in determining how a tumor responds to various treatments.

Next, we analyzed the correlation between CISD1 and immune cell infiltration in the TME, including B cells, CD4^+^ T cells, CD8^+^ T cells, neutrophils, macrophages, and dendritic cells. As is shown in Figure 7D, CISD1 was significantly and positively correlated with immune cell infiltration in KIRC, KIPAN, PCPG, PRAD, KICH, OV, BRCA, PAAD, SKCM-P (primary SKCM), LIHC, BLCA, and HNSC, suggesting that CISD1 may play a role in facilitating an effective immune response against the tumor, and tumors with higher CISD1 expression might respond better to immunotherapies in these cancers. In contrast, CISD1 was significantly and negatively correlated with immune cells infiltration in STES, ESCA, STAD, GBMLGG, DLBC, THYM, and TGCT, suggesting that CISD1 might contribute to immune suppression and tumor evasion, and tumors with high CISD1 expression may be more resistant to immunotherapies in these cancers.

Taken together, these results suggest that CISD1 may be associated with a more active immune response and potentially better outcomes in those positive correlation cancers, which makes CISD1 a potential marker of immunogenic tumors that may respond well to immunotherapy. In contrast, CISD1 might be involved in immune evasion and tumor progression in those negative correlation cancers, which makes CISD1 a potential target for enhancing immune infiltration and improving the effectiveness of cancer treatments.

### CISD1 correlates with immune checkpoint blockade proteins and is a potential biomarker for tumor immunotherapy

**Figure 8:**
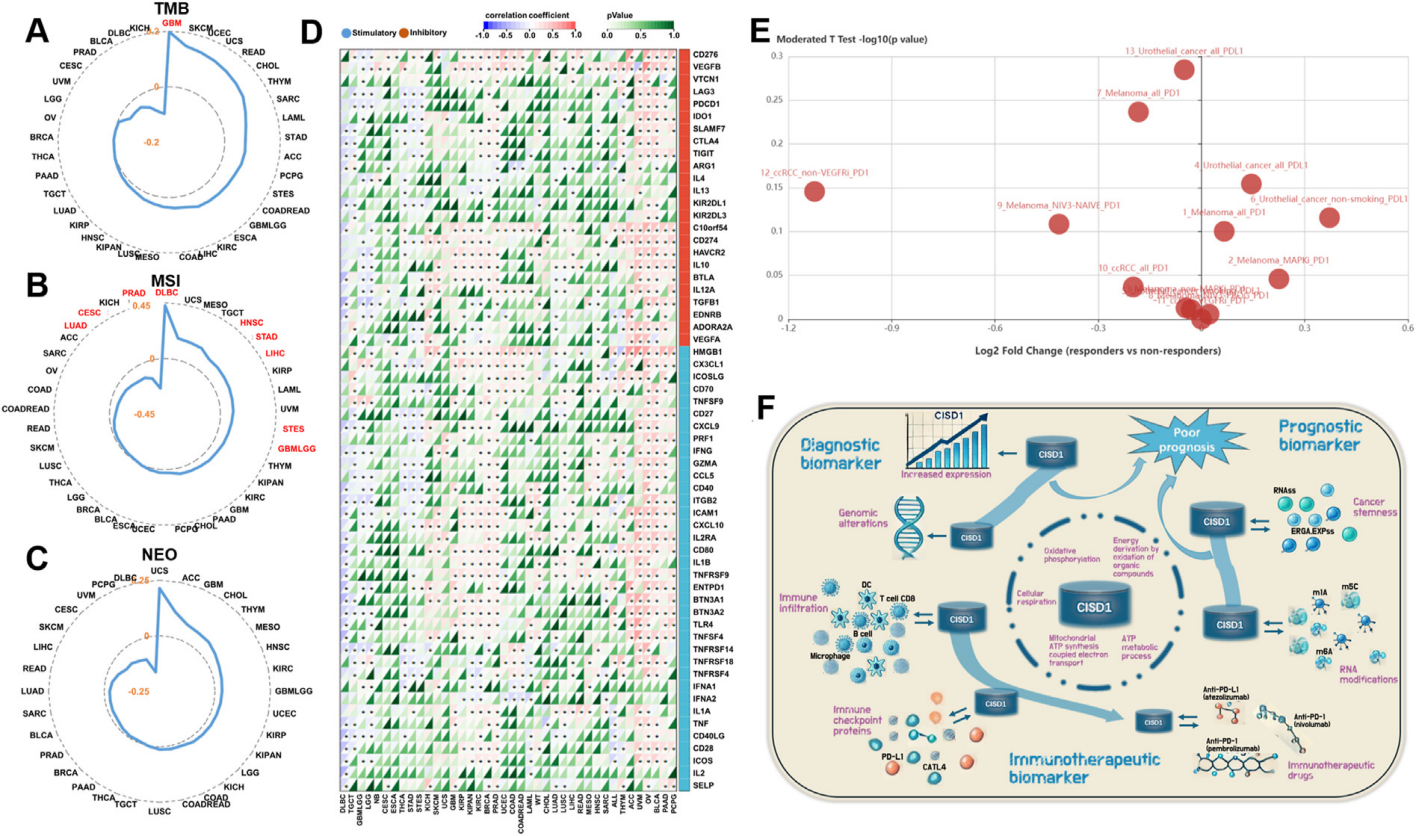
Correlation analysis between CISD1 and immune checkpoint blockade proteins. **(A**–**C)** The radar plots generated from SangerBox illustrate the correlation between CISD1 expression and tumor mutation burden (TMB) (A), microsatellite instability (MSI) (B), and neoantigen burden (NEO) (C) across various cancers. The correlation coefficients are shown, with cancer types labeled around the plot perimeter. Significant correlations (*p* < 0.05) are highlighted in red, and the blue lines indicate the correlation values. Negative correlation coefficients are presented inside the plots. **(D)** The heatmap generated from SangerBox shows the correlation between CISD1 expression and various immune checkpoint blockade-related genes, including PD1, PD-L1, and CTLA4, across multiple cancer types. The color gradient represents correlation coefficients, with red indicating positive correlations and blue indicating negative correlations. Significant correlations are marked by stars (∗), and dark green means no significant. **(E)** The scatter plot generated from TISIDB shows the log_2_ fold change between responders and non-responders to immune checkpoint blockade therapy (*e.g.*, PD-1, PD-L1) in various cancer types. The position on the *X*-axis represents the fold change, while the *Y*-axis shows the moderated *t*-test *p*-values. **(F)** CISD1 is a diagnostic, prognostic, and immunotherapeutic biomarker in multiple cancers. This picture simply summarizes the evidence that CISD1 is a reliable and promising biomarker. CISD1 plays a critical role in cellular energetics; it participates in many biological processes related to energy and metabolism. Its expression levels are elevated in multiple cancers, and it undergoes genetic alterations in various cancers, which enable it to aid in cancer early diagnosis. Patients with increased expression levels of CISD1 have relatively lower survival rates; moreover, its positive correlation with tumor stemness indices and RNA modifications indicates that it can be used to predict prognosis. It is positively correlated with tumor infiltration and immune checkpoint genes, and it exhibits higher expression levels in patients who respond to tumor immunotherapy, suggesting that it can be used to predict the outcome of cancer immunotherapy.

The above results suggest that CISD1 is associated with immunotherapies. Thus, we analyzed the correlation between CISD1 and TMB, MSI, or neoantigen burden (NEO), to help understand if CISD1 is associated with generating neoantigens. Specifically, high TMB and NEO load in MSI tumors favor strong antitumor immune responses.^46^^,^^47^ Data was downloaded from SangerBox and analyzed, and Figure 8A shows that CISD1 was significantly and positively correlated with TMB in GBM, suggesting that higher CISD1 expression is associated with a higher number of mutations. This suggests that CISD1 might be a potential biomarker for predicting the efficacy of immunotherapy in GBM. CISD1 also showed significant positive correlation with MSI in DLBC, HNSC, STAD, LIHC, STES, and GBMLGG (Fig. 8B), suggesting that patients with high CISD1 expression are more likely to benefit from immunotherapies in these cancers. In contrast, CISD1 showed a significant negative correlation with MSI in LUAD, CESC, and PRAD (Fig. 8B), suggesting that patients with high CISD1 expression are less likely to respond to immunotherapies in these cancers. However, CISD1 showed no significant correlation with NEO in all tested cancers (Fig. 8C).

Next, we analyzed the correlation between CISD1 and immune checkpoint blockade proteins, as they are targets for immune checkpoint blockades. CISD1 was significantly and positively correlated with most of the immune checkpoint proteins in PCPG, PAAD, BLCA, OV, UVM, ACC, THYM, HNSC, LUSC, LUAD, PRAD, BRCA, KIRC, KIPAN, KIRP, GBM, and SKCM (Fig. 8D), suggesting that CISD1 might play a role in the immune evasion of tumors, making CISD1 an important biomarker for both prognosis and immunotherapies. Thus, patients with high CISD1 expression may benefit from checkpoint inhibitor therapies in these cancers. Conversely, CISD1 was significantly and negatively correlated with most of the immune checkpoint proteins in STES, STAD, THCA, LGG, GBMLGG, TGCT, and DLBC (Fig. 8D), suggesting that CISD1 might be linked to a more active immune environment within the TME of these tumors. Understanding correlations between CISD1 and immune-related genes can also provide insight into how tumors with high CISD1 expression might respond to immunotherapy. Chemokines help recruit immune cells,^48^ and MHC genes play crucial roles in antigen presentation to immune cells, which is essential for initiating an immune response against tumor cells.^49^ Figure S4A showed that CISD1 was positively correlated with immune chemokines in BLCA, BRCA, CESC, GBM, HNSC, LUSC, MESO, OV, SARC, THCA, and UCS, suggesting that higher CISD1 expression might promote chemokine expression, potentially influencing immune cell recruitment. Figure S4B showed that CISD1 was positively correlated with MHC genes in BLCA, BRCA, CESC, CHOL, GBM, HNSC, KIRC, KIRP, LIHC, LUSC, MESO, OV, SARC, THCA, and UCS, suggesting that high CISD1 expression is associated with up-regulated MHC gene expression, potentially enhancing antigen presentation and immune recognition.

Furthermore, we wondered if CISD1 expression differed between checkpoint blockade (*e.g.*, anti-PDL1 and anti-PD1) responders and non-responders. To investigate this, we used TISIDB, an integrated repository portal for tumor-immune system interactions, which collects data sets of transcriptomic and genomic profiling of pre-treated tumor biopsies from immunotherapeutic responders and non-responders.^31^ We used the TISIDB portal to analyze the relationship between CISD1 expression and immunotherapy response, and found that immunotherapeutic responders had higher CISD1 expression than non-responders in Melanoma_all_PD1 (pembrolizumab and nivolumab), Melanoma_MAPKi_PD1 (pembrolizumab and nivolumab), Urothelial_cancer_all_PDL1 (atezolizumab), and Urothelial_cancer_non-smoking_PDL1 (atezolizumab) (Fig. 8E and Table 1), suggesting that CISD1 is up-regulated in these responders and might serve as a prognostic marker in these cancers; while immunotherapeutic responders had lower CISD1 expression than non-responders in Melanoma_all_PD1 (nivolumab), Melanoma_NIV3-NAÏVE_PD1 (nivolumab), ccRCC_all_PD1 (nivolumab), ccRCC_non-VEGFRi_PD1 (nivolumab), and Urothelial_cancer_all_PDL1 (atezolizumab) (Fig. 8E and Table 1), suggesting that CISD1 is down-regulated in these responders and may be linked with resistance to immunotherapy.

**Table 1:** The difference in CISD1 expression between immunotherapeutic responders and non-responders.

CISD1	No.	PMID	Cancer type	Group	Drug	#Responders	#Non-responders	Log_2_(fold change)	*p* value
Up-regulated	1	26997480	Melanoma	All	Anti-PD-1 (pembrolizumaband nivolumab)	14	12	0.066	0.794
	2	26997480	Melanoma	MAPKi	Anti-PD-1 (pembrolizumaband nivolumab)	6	5	0.225	0.9
	4	28552987	Urothelial cancer	All	Anti-PD-L1 (atezolizumab)	9	16	0.145	0.701
	6	28552987	Urothelial cancer	Non-smoking	Anti-PD-L1 (atezolizumab)	4	7	0.372	0.766
Down-regulated	7	29033130	Melanoma	All	Anti-PD-1 (nivolumab)	26	23	−0.183	0.58
	9	29033130	Melanoma	NIV3-NAIVE	Anti-PD-1 (nivolumab)	11	12	−0.414	0.779
	10	29301960	Clear cell renalcell carcinoma	All	Anti-PD-1 (nivolumab)	4	8	−0.198	0.919
	12	29301960	Clear cell renalcell carcinoma	Non-VEGFRi	Anti-PD-1 (nivolumab)	2	8	−1.124	0.715
	13	29443960	Urothelial cancer	All	Anti-PD-L1 (atezolizumab)	68	230	−0.05	0.519

Taken together, these results highlight the potential importance of CISD1 in cancer progression and treatment, particularly in the context of the immune response and tumor microenvironment. Given its associations with immune cell infiltration and immunotherapy response, CISD1 can be explored as a potential therapeutic target and/or a biomarker for predicting treatment outcomes in cancer patients.

## Discussion

CISD1 is a multifunctional protein localized to the outer membrane of mitochondria.^9^ Up-regulation of CISD1 was reported in various cancer types,^19^^,^^20^^,^^50^ and CISD1 was shown to play crucial roles in tumor progression, resistance to apoptosis, and poor prognosis,^9^^,^^14^ making it an interesting topic of research in understanding carcinogenesis and developing therapeutic strategies. This study is the first comprehensive pan-cancer bioinformatics analysis to determine the functions of CISD1 in multiple cancers using public patient databases, and demonstrate that CISD1 is a potential diagnostic, prognostic and immunotherapeutic biomarker for multiple cancers.

The claim above is supported by the following lines of evidence. First, CISD1 can be a diagnostic biomarker to aid early detection of various cancers. Gene expression level alterations often serve as important diagnostic biomarkers for the early detection of cancer. For example, increased prostate-specific antigen (PSA) protein serves as a biomarker for early detection of prostate cancer.^51^ Elevated alpha-fetoprotein (AFP) levels are used as a diagnostic marker for liver cancer and to monitor disease progression.^52^ The immune checkpoint receptor programmed cell death ligand 1 (PD-L1) is overexpressed in several cancers and can be used to predict response to immune checkpoint inhibitors.^53^ In this study, we found that expression levels of CISD1 were dramatically dysregulated in various cancers. GEPIA2.0 online analysis of TCGA datasets showed that mRNA levels of CISD1 were significantly up-regulated in 22 of 33 total cancer types analyzed (Fig. 1C); and the following THPA analysis showed that protein expression levels of CISD1 were also significantly increased in multiple cancers (Fig. 2). These results demonstrate that CISD1 expression is significantly associated with various cancer types. Elevated levels of CISD1 in specific cancers indicate its possible oncogenic role in tumor growth, and oncogenes play multiple, interconnected roles in cancer progression by driving cell proliferation and promoting metastasis.^54^ Indeed, CISD1 is also associated with cancer stages development, as its mRNA expression levels are increased with the progress of the cancer stages and metastasis in various cancers (Fig. S1). Our analysis is consistent with previous studies that have showed CISD1 is up-regulated in several cancers.^13^^,^^15^^,^^16^ Gene alterations can also serve as biomarkers to aid diagnosis of cancers. For instance, mutations in the BRCA1 and BRCA2 genes are strongly associated with an increased risk of breast and ovarian cancers, and genetic testing for BRCA1 and BRCA2 mutations help identify individuals at high risk.^55^ Mutations in TP53 can lead to a loss of its tumor suppressive functions, contributing to cancer development, and early detection of these mutations can lead to early intervention for multiple cancer types.^56^ In our study, CISD1 showed frequent gene mutations, amplifications, and deletions in multiple cancers (Fig. 3A–D). Missense mutations were the most frequent type of CISD1 gene mutations in various cancers (Fig. 3D), highlighting that the mutations of CISD1 could be targets for diagnostic assays. Copy number alterations were the most common genomic change in most of the cancers (Fig. 3A), and cancers with higher rates of CISD1 amplifications had a higher expression level of CISD1, which further confirms that CISD1 is an oncogene in multiple cancers (Fig. 3E). Moreover, expression of CISD1 was strongly associated with somatic mutations, such as TP53 mutations (Fig. 3F). All these genomic alteration profiles indicate that CISD1 is a promising diagnostic biomarker.

Second, CISD1 can be a prognostic biomarker for cancer patients. The expression level of certain oncogenes is often correlated with overall survival rates in cancer patients, and tumors with high oncogene activity often exhibit shorter overall survival and disease-free survival.^57^^,^^58^ Some mutations can also be directly associated with patient survival. For instance, mutations in tumor suppressor genes like TP53 are often associated with a worse prognosis, as they may lead to more aggressive tumor behavior and poorer overall outcomes.^56^ Our study validated that CISD1 could be a reliable biomarker for cancer prognosis, as patients with higher expression levels of CISD1 had a lower survival probability from the overall survival analysis in multiple cancers (Fig. 4). Moreover, patients with higher expression levels of CISD1 had a higher risk of death from analyses of disease-specific survival, disease-free survival, and progression-free survival (Fig. S2). Our results are also consistent with previous reports that CISD1 is a prognostic biomarker in several cancers.^18^^,^^19^ This reproducible and significant correlation between CISD1 expression and patient survival across various cancers strengthens its use as a prognostic biomarker in clinical settings. Stemness-related signatures can also be used to predict patient outcomes. High levels of stemness in tumors are often associated with a poor prognosis, as these tumors may be more aggressive and likely to metastasize.^59^ Our study revealed that CISD1 was significantly and positively correlated with stemness indices in multiple cancers (Fig. 5A–F), indicating that CISD1 is involved in promoting or maintaining stem cell-like properties in cancer cells. Stemness is a key feature of cancer aggressiveness, treatment resistance, and metastatic potential. The strong correlation between CISD1 and stemness indices underscores its potential role in driving tumor aggressiveness and therapeutic resistance, and high CISD1 expression may serve as an indicator of tumors with enriched cancer stem cell populations, predicting more aggressive disease and reduced therapeutic efficacy. By modulating cancer stem cell characteristics, CISD1 not only contributes to tumor aggressiveness but also presents as a promising target for innovative therapies aimed at eradicating cancer stem cell-driven disease. Therefore, the correlation could have profound implications for prognosis.^60^ Indeed, patients with high expression of CISD1 (Fig. 1) and high levels of stemness (Fig. 5) have worse prognosis (Fig. 4), such as BRCA, LIHC, LUAD, SKCM, and THYM in our study. Given the association between cancer stem cell-related stemness indices and poor patient outcomes, the correlation with CISD1 highlights its potential as a prognostic biomarker. Targeting CISD1 in cancers with high stemness signatures may offer a promising therapeutic avenue. In addition to stemness, CISD1 is also associated with epigenetic regulation in multiple cancers in our study. Figure 5G showed strong positive correlations between CISD1 and RNA modifications like m1A, m5C, and m6A, indicating that CISD1 could serve as a biomarker for cancer prognosis, as high CISD1 expression, along with altered RNA modifications, might be associated with more aggressive tumor behavior and could predict poor outcomes in certain cancers including KICH, BRCA, LIHC, LUAD, and SKCM.

Understanding the biological functions and pathways associated with CISD1 can potentiate its prognostic value. Our study showed that 26 genes are coexpressed with CISD1 across five types of cancer that have high expression levels of CISD1, high stemness signatures, high levels of RNA modifications, and worse patient survival (Fig. 6A), and 20 of them are highly expressed across a wide range of cancers (Fig. 6B). GO and KEGG enrichment analysis showed that these CISD1 coexpression genes were involved in critical pathways in energy metabolism including oxidative phosphorylation, mitochondrial electron transport, and cellular respiration (Fig. 6C–G). These pathways are often altered in cancer cells to support rapid growth and survival. Our findings are consistent with the previous report that CISD1 plays critical roles in bioenergetics.^10^ Indeed, CISD1 showed a negative correlation with age in multiple cancers (Fig. S3A), indicating that CISD1 expression is higher in younger individuals and tends to decrease in older individuals. The decline in CISD1 expression with age may reflect its role in maintaining cellular bioenergetics.

Third, CISD1 can be an immunotherapeutic biomarker indicating the likelihood of a patient responding to immunotherapy. Immunotherapy has revolutionized cancer treatment by harnessing the immune system to target and destroy cancer cells. Unlike traditional therapies, immunotherapy provides a more personalized approach, with varying outcomes based on patient-specific factors. Certain factors have been identified as immunotherapeutic biomarkers to predict the outcomes of immunotherapy in cancer treatment. For example, high expression of PD-L1 in tumors often correlates with better responses to programmed death-1 (PD-1)/PD-L1 inhibitors such as nivolumab or pembrolizumab.^61^ High TMB or MSI has been reported to link to better response to immune checkpoint inhibitors.^62^^,^^63^ Further analysis showed that CISD1 expression was positively correlated with immune cells in multiple cancers (Fig. 7D), indicating that tumors with high expression of CISD1 have high immune infiltration. The degree of immune infiltration in a tumor is a critical factor in determining the success of immunotherapy treatments. Indeed, CISD1 is significantly positively correlated with TMB and MSI in several cancers (Fig. 8A, B), indicating that patients with high expression of CISD1, together with high TMB and MSI, can benefit from immunotherapy, as a high TMB or MSI has been linked to better response to immune checkpoint inhibitors.^62^^,^^63^ Importantly, immune checkpoint analysis showed that CISD1 was significantly and positively correlated with immune checkpoint genes such as programmed cell death 1 (PDCD-1; PD-1 coding gene), CD274 (PD-L1 coding gene), or cytotoxic T-lymphocyte associated protein 4 (CTLA4) in multiple cancers (Fig. 8D), indicating that tumors with high expression of CISD1 have a high expression of immune checkpoint proteins, which serve as targets for immune checkpoint blockades. This finding aligns with a recent study identifying CISD1 as a ferroptosis-related gene in breast cancer, which similarly showed significant correlations between CISD1 and immune checkpoints, including PDCD-1, CTLA4, and lymphocyte activating 3 (LAG3).^64^ Although no direct experimental evidence has yet established a regulatory relationship between CISD1 and these immune checkpoints, CISD1's association with mitophagy and hypoxia, alongside evidence linking immune checkpoints to mitophagy and tumor hypoxia, suggests an important connection among the three. For example, while inhibition of CISD1 induces mitophagy mediated by Parkin,^65^^,^^66^ Parkin deficiency (a key mitophagy mediator) reduces mitophagy in the liver, increases PD-1 and CTLA4 expression, creates an immunosuppressive microenvironment, and promotes hepatocarcinogenesis.^67^ Moreover, PD-L1 expression increases under tumor hypoxia, contributing to immune evasion and tumor progression.^68^ Notably, tumor samples from breast cancer patients who responded to combined immunotherapy with immune checkpoint inhibitors and paclitaxel displayed significant PD-L1 distribution in mitochondria.^69^ Taken together, these findings support our speculation that high CISD1 expression may suppress mitophagy, help counteract hypoxia, and maintain a growth advantage for tumor cells, while simultaneously up-regulating immune checkpoints to promote immune evasion. As these elevated immune checkpoints serve as effective immunotherapeutic targets, CISD1 overexpression could be a reliable biomarker for immunotherapy. Furthermore, using published clinical data, we found that CISD1 expression was higher in immunotherapy responders versus non-responders (Fig. 8E). Thus, increased expression of CISD1 can be used to predict response to immune checkpoint inhibitors in cancers. These results strongly support that CISD1 can be a great immunotherapeutic biomarker.

Studies have shown that CISD1 regulates the levels of iron and reactive oxygen species in cancer cells.^8^ It also inhibits mitophagy, apoptosis, and ferroptosis in cancer cells, highlighting its central role in promoting cancer cell proliferation, supporting tumor growth, and facilitating metastasis.^8^ Our pan-cancer analysis further confirms that CISD1 is significantly overexpressed in various cancer types, conferring a survival advantage to cancer cells. These findings underscore the potential of CISD1 as a highly effective target in cancer therapy. Since the function of mitoNEET (CISD1) depends on the relative instability of its [2Fe–2S] cluster and cluster transfer reactions,^8^ drugs that stabilize its iron-sulfur cluster could serve as promising anticancer agents, with pioglitazone being one such example. MitoNEET (CISD1) was first identified as a binding target for pioglitazone,^70^ and pioglitazone binding stabilizes the iron-sulfur cluster of mitoNEET and prevents its transfer.^71^^,^^72^ Treatment of breast cancer cells with pioglitazone leads to increased mitochondrial iron and reactive oxygen species levels, reducing the cells' tolerance to oxidative stress and subsequently inducing cancer cell death.^13^^,^^73^ In human hepatocellular carcinoma cells, pioglitazone inhibits mitochondrial iron uptake and lipid peroxidation, further preventing ferroptosis by stabilizing the CISD1 iron-sulfur cluster.^74^ Building on the success of pioglitazone, novel compounds have been developed to target CISD1 in cancer. For instance, mitoNEET ligand-1 (NL-1), a derivative of pioglitazone designed to target drug-resistant leukemia cells, demonstrates potent anti-cancer activity by stabilizing mitoNEET's iron-sulfur cluster.^16^ Another promising compound, NTS-01, specifically binds to human mitoNEET protein, stabilizes its [2Fe–2S] cluster under oxidative conditions *in vitro*, and induces mitochondrial fission, significantly reducing ovarian cancer cell proliferation.^75^ Our pan-cancer analysis highlights CISD1 as not only a reliable biomarker but also a highly promising anti-cancer target due to its overexpression in multiple cancer types. The development of novel therapeutics targeting CISD1's iron-sulfur cluster or modulating its protein expression holds great potential for improving cancer outcomes.

In addition to CISD1 up-regulation in the vast majority of cancers analyzed, we observed that CISD1 expression was down-regulated in six types of cancer (Fig. 1C), suggesting that it may play a different role in these cancers, potentially acting as a tumor suppressor. Cancer is highly heterogeneous, meaning that the genetic mutations, signaling pathways, and microenvironments vary significantly between cancers, and this heterogeneity can influence how a gene functions.^76^ Genes can play opposing roles in different cancers due to the unique molecular and cellular contexts of each cancer type. For example, the well-known tumor suppressor gene TP53 can lose its tumor-suppressive function and even gain oncogenic functions in certain cancer types.^56^ Depending on the cancer type, NOTCH can act as an oncogene (*e.g.*, in T-cell acute lymphoblastic leukemia) or as a tumor suppressor (*e.g.*, in skin cancer).^77^ Furthermore, CISD1's expression in some cancers was negatively correlated with tumor indices (Fig. 5A–E), RNA modification enzymes (Fig. 5G), immune cell infiltration (Fig. 7D), or immune checkpoint proteins (Fig. 8D). The relationship between CISD1 and these cancer-related factors can differ across cancer types due to variations in tumor genetic and epigenetic context and tumor microenvironment, as each cancer type has a unique set of genetic alterations and epigenetic modifications that can modulate how a gene interacts with other molecules and signaling pathways,^78^ and tumor microenvironment can alter gene function and expression, leading to different effects of a gene.^79^ A dual role of CISD1 in cancer is not unusual, as many genes exhibit context-dependent functionality.^56^^,^^77^ This flexibility is shaped by factors such as the specific cancer type, tumor microenvironment, and genetic background. Even with opposing expression levels in different cancers, CISD1 can still provide meaningful diagnostic, prognostic, or predictive information from our findings. CISD1 serving as a biomarker provides valuable information relevant to specific clinical outcomes in our study.

We acknowledge that this study relies on computational analyses based on publicly available datasets, which, while robust and widely used, have inherent limitations. The lack of experimental validation of our findings is a significant limitation, as computational predictions require laboratory-based confirmation to establish causal relationships and functional mechanisms. Furthermore, the dataset heterogeneity, including variations in data processing and normalization across platforms, may introduce biases that could influence the results. Despite these limitations, our integrative analysis provides valuable insights into the potential roles of CISD1 in cancer biology and identifies it as a promising biomarker and therapeutic target. This systematic pan-cancer analysis lays a strong foundation for further exploring the biological functions of CISD1 in cancers. Future studies will focus on experimental validation of CISD1's role in tumor aggressiveness, stemness, and immunotherapy, and exploration of its molecular mechanisms through *in vitro* and *in vivo* experiments.

In summary, our systematic pan-cancer analysis of CISD1, for the first time, shows that its expression is significantly altered in the vast majority of cancers analyzed. Additionally, it undergoes mutations in multiple cancers, suggesting its potential as a diagnostic biomarker for cancers. Moreover, cancer patients with significantly altered expression of CISD1 have lower survival rates and worse prognoses, and its expression is significantly correlated with tumor stemness indices in multiple cancers, highlighting its importance prognostic prediction as a crucial prognostic biomarker. More importantly, it can serve as a biomarker for predicting cancer patients' response to immunotherapy, as its expression is not only significantly correlated with TMB and MSI but also with immune checkpoints, and its expression is altered in tumors of patients who respond to immunotherapy. In conclusion, all these findings strongly demonstrate that CISD1 is a reliable and promising diagnostic, prognostic, and immunotherapeutic biomarker in multiple cancers (Fig. 8F).

## CRediT authorship contribution statement

**Caiyue Li:** Writing – review & editing, Writing – original draft, Visualization, Validation, Software, Resources, Methodology, Formal analysis, Data curation, Conceptualization. **Zhipin Liang:** Writing – review & editing. **Gabrielle Vontz:** Writing – review & editing. **Connor Kent:** Writing – review & editing. **Wenbo Ma:** Writing – review & editing, Methodology. **Lei Liu:** Writing – review & editing. **Riya Dahal:** Writing – review & editing. **Jovanny Zabaleta:** Writing – review & editing. **Guoshuai Cai:** Writing – review & editing, Methodology. **Jia Zhou:** Writing – review & editing, Resources, Project administration, Funding acquisition. **Huangen Ding:** Writing – review & editing, Resources, Project administration, Funding acquisition. **Qiang Shen:** Writing – review & editing, Supervision, Resources, Project administration, Funding acquisition, Conceptualization.

## Funding

This project was supported by the Louisiana State University Health Sciences Center (LSUHSC) Startup funds (New Orleans, Louisiana, USA) (to Q.S.), Louisiana State University (LSU) Interinstitutional Cancer Research Funding Initiative (Louisiana, USA) (CCRI; to H.D. and Q.S.), Louisiana Cancer Research Center (LCRC) Strategic Investment in Translational Research Awards (Louisiana, USA) (to Q.S. and H.D.), the John D. Stobo, M.D. Distinguished Chair Endowment (USA) (to J.Z.), and the Edith & Robert Zinn Chair Endowment in Drug Discovery (USA) (to J.Z.).

## Conflict of interests

The authors declared no conflict of interests.

## Figures and Tables

**figs1:**
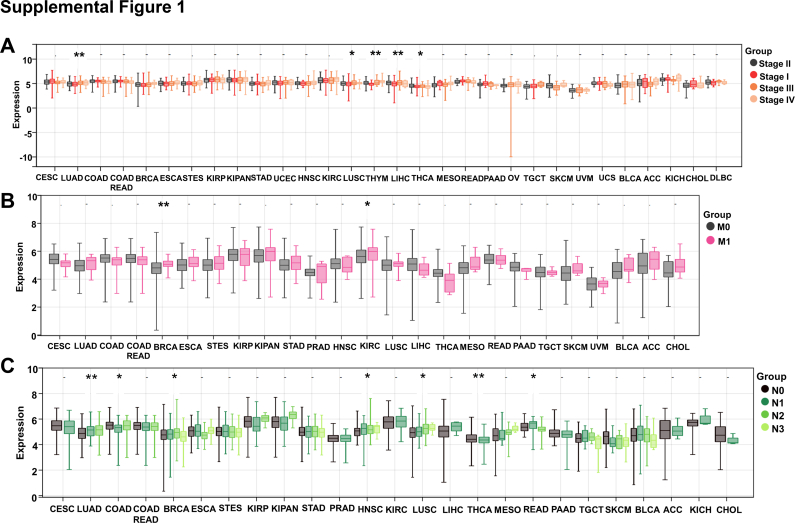


**figs2:**
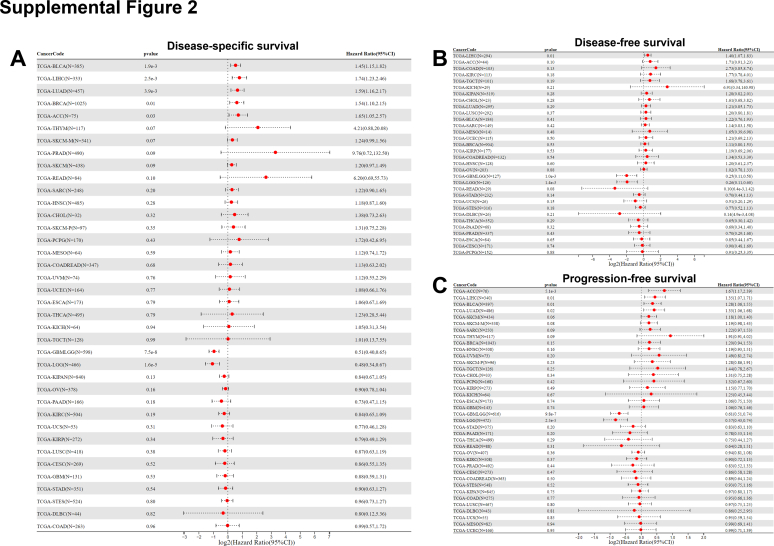


**figs3:**
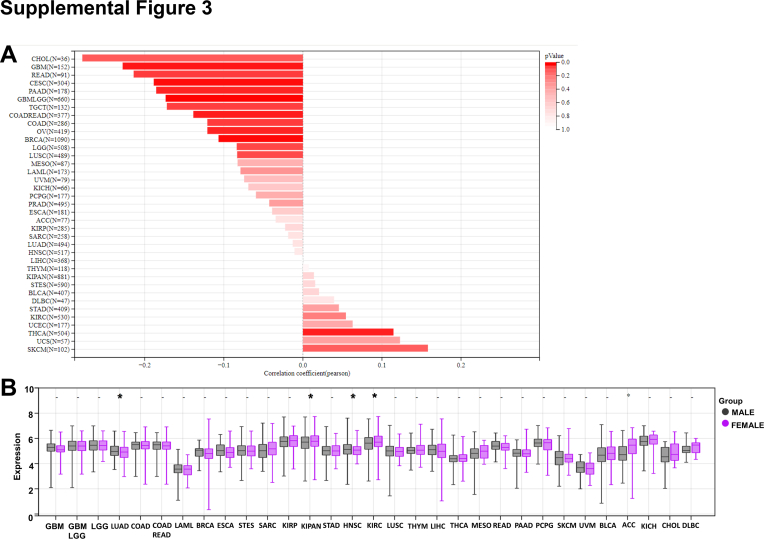


**figs4:**