# Supplementary material for: Exploring CISD1 as a multifaceted biomarker in cancer: Implications for diagnosis, prognosis, and immunotherapeutic response
Source: Genes Dis. 2025 May 8;12(6):101677. doi: 10.1016/j.gendis.2025.101677 (PMC12359157; doi:10.1016/j.gendis.2025.101677)
Supplement: Multimedia component 1 [file mmc1.docx]

**Table S1** Abbreviation for various cancers.

| **Study Abbreviation** | **Study Name** |
| --- | --- |
| ACC | Adrenocortical carcinoma |
| ALL | Acute Lymphoblastic Leukemia |
| BLCA | Bladder Urothelial Carcinoma |
| BRCA | Breast invasive carcinoma |
| CESC | Cervical squamous cell carcinoma and endocervical adenocarcinoma |
| CHOL | Cholangiocarcinoma |
| COAD | Colon adenocarcinoma |
| COADREAD | Colon adenocarcinoma/Rectum adenocarcinoma Esophageal carcinoma |
| DLBC | Lymphoid Neoplasm Diffuse Large B-cell Lymphoma |
| ESCA | Esophageal carcinoma |
| GBM | Glioblastoma multiforme |
| GBMLGG | Glioma |
| HNSC | Head and Neck squamous cell carcinoma |
| KICH | Kidney Chromophobe |
| KIPAN | Pan-kidney cohort (KICH+KIRC+KIRP) |
| KIRC | Kidney renal clear cell carcinoma |
| KIRP | Kidney renal papillary cell carcinoma |
| LAML | Acute Myeloid Leukemia |
| LGG | Brain Lower Grade Glioma |
| LIHC | Liver hepatocellular carcinoma |
| LUAD | Lung adenocarcinoma |
| LUSC | Lung squamous cell carcinoma |
| MESO | Mesothelioma |
| NB | Neuroblastoma |
| OV | Ovarian serous cystadenocarcinoma |
| PAAD | Pancreatic adenocarcinoma |
| PCPG | Pheochromocytoma and Paraganglioma |
| PRAD | Prostate adenocarcinoma |
| READ | Rectum adenocarcinoma |
| SARC | Sarcoma |
| SKCM | Skin Cutaneous Melanoma |
| SKCM-M | Metastatic Skin Cutaneous Melanoma |
| SKCM-P | Primary Skin Cutaneous Melanoma |
| STAD | Stomach adenocarcinoma |
| STES | Stomach and Esophageal carcinoma |
| TGCT | Testicular Germ Cell Tumors |
| THCA | Thyroid carcinoma |
| THYM | Thymoma |
| UCEC | Uterine Corpus Endometrial Carcinoma |
| UCS | Uterine Carcinosarcoma |
| UVM | Uveal Melanoma |
| WT | High-Risk Wilms Tumor |

**Figure S1** CISD1 expression analysis in various tumor stages, metastasis status, and lymph node involvement across cancer types. **(A)** Boxplot shows CISD1 expression levels across different cancer types based on tumor stage (stages I-IV). Each box represents the median and distribution of expression for each cancer type at a specific stage. **(B)** Boxplot of CISD1 expression levels in relation to metastasis status (M0 *vs*. M1) across various cancers. M0 (no metastasis) is shown in gray, and M1 (presence of metastasis) is shown in pink. **(C)** Boxplot of CISD1 expression across different levels of lymph node involvement (N0-N3) in various cancers. All results were obtained from SangerBox. Significant differences in CISD1 expression are marked by asterisks (^*^*P* < 0.05, ^**^*P* < 0.01).

**Figure S2** Correlation analysis between CISD1 expression and survival outcomes in different cancers. **(A)** The forest plot shows the relationships between CISD1 expression and disease-specific survival across multiple cancer types. Hazard ratios (HR) with 95% confidence intervals (CI) are displayed, where an HR greater than 1 suggests an association with poorer survival. **(B)** The forest plot displays the correlation between CISD1 expression and disease-free survival. Log_2_ (hazard ratio) and 95% confidence intervals are plotted for each cancer type. **(C)** The forest plot illustrates the correlation between CISD1 expression and progression-free survival. The log_2_ (hazard ratio) is shown. All results were obtained from SangerBox.

**Figure S3** Correlation analysis between CISD1 expression and age or gender across multiple cancer types. **(A)** The bar plot shows the Pearson correlation coefficients between CISD1 expression and patient age across various cancer types. Each bar represents the strength of the correlation, with red shades indicating different *P*-values (from light to dark, where darker indicates higher significance). **(B)** The boxplot compares CISD1 expression between male and female patients across multiple cancers. Male patients are represented by gray boxes, and female patients are represented by purple boxes. Statistical significance between genders is marked with asterisks (^*^*P* < 0.05). Each box represents the median and distribution of CISD1 expression levels for each cancer type. All results were obtained from SangerBox.

**Figure S4** Correlation analysis between CISD1 and immune-related genes across multiple cancers. **(A)** The heatmap shows the correlation between CISD1 expression and various chemokine genes across different cancer types. **(B)** The heatmap displays the correlation between CISD1 expression and major histocompatibility complex (MHC) genes across different cancer types. Correlation coefficients are color-coded, with red indicating positive correlations and blue indicating negative correlations. The scale bar from −1 to 1 reflects the strength of the correlation. All results were obtained from TISIDB.
